# Sustainable Biocomposites Reinforced with Waste Artichoke Stem and Modified Soybean Oil: Multifunctional Properties and ANN-Based Prediction

**DOI:** 10.3390/polym18172101

**Published:** 2026-08-29

**Authors:** Muhammet Aydın, Maruf Hurşit Demirel, Ercan Aydoğmuş

**Affiliations:** 1 Department of Mechatronics Engineering, Faculty of Engineering, Fırat University, Elazığ 23119, Türkiye; 2 Department of Basic Sciences, Faculty of Pharmacy, Fırat University, Elazığ 23119, Türkiye; 3 Department of Chemical Engineering, Faculty of Engineering, Fırat University, Elazığ 23119, Türkiye; 4 Department of Materials Science and Engineering, University of North Texas, Denton, TX 76203, USA

**Keywords:** polyurethane-based biocomposites, waste artichoke stem, modified soybean oil, artificial neural network, training algorithms, multifunctional properties

## Abstract

Sustainable polyurethane-based biocomposites (PUBs) reinforced with waste artichoke stem (WAS) and modified soybean oil (MSO) provide a promising approach for agricultural-waste valorization and the development of multifunctional polymeric materials. In this study, 48 PUB formulations are prepared by systematically varying WAS content from 0.0 to 3.5 wt.% and MSO content from 0 to 5 wt.%. The dielectric constant, thermal conductivity, Shore A hardness, bulk density, tensile strength, and elongation at break are experimentally evaluated. Across the investigated formulations, the dielectric constant, thermal conductivity, Shore A hardness, bulk density, tensile strength, and elongation at break range from 1.10 to 1.56, 0.024 to 0.036 W m^−1^ K^−1^, 9.5 to 43.0, 34.0 to 67.0 kg m^−3^, 115 to 297 kPa, and 62 to 176%, respectively. A multi-output artificial neural network (ANN) model is developed in MATLAB using WAS and MSO contents as input variables and the six experimentally determined properties as simultaneous outputs. The ANN architecture consists of two input neurons, one hidden layer with ten neurons, and six output neurons. Levenberg–Marquardt (LM), Bayesian Regularization (BR), and Scaled Conjugate Gradient (SCG) algorithms are comparatively evaluated using 42 samples for model development and six independent samples for external validation. The results demonstrate that the ANN successfully captures the nonlinear relationships between formulation variables and the investigated multifunctional properties. Among the evaluated training algorithms, BR provides the most accurate and robust predictive performance, followed by LM, whereas SCG exhibits comparatively lower prediction accuracy. The proposed experimental–computational framework enables reliable simultaneous prediction of the electrical, thermal, physical, and mechanical properties of PUBs and provides an efficient strategy for reducing experimental effort and accelerating the formulation and performance assessment of sustainable biocomposites.

## 1. Introduction

Polymer-based composite materials have become indispensable in modern engineering applications, including transportation, aerospace, civil infrastructure, electronics, marine systems, and biomedical devices, owing to their high specific strength, lightweight nature, corrosion resistance, and excellent design versatility. Nevertheless, the extensive dependence of conventional composites on petroleum-derived polymer matrices and synthetic reinforcements has raised significant environmental concerns associated with fossil resource depletion, greenhouse gas emissions, high manufacturing energy demand, and the accumulation of persistent composite waste after service life. These challenges have accelerated the transition toward sustainable material systems that comply with the principles of the circular economy and green manufacturing. In this context, bio-based polymers and lignocellulosic reinforcements obtained from renewable resources and agricultural residues have emerged as promising alternatives for developing environmentally benign composite materials. The valorization of agricultural by-products not only reduces waste disposal and environmental pollution but also creates value-added materials with competitive mechanical and functional properties while lowering production costs. Consequently, natural fiber-reinforced biocomposites have attracted increasing scientific and industrial interest as sustainable substitutes for conventional polymer composites, offering a practical pathway toward resource-efficient materials with reduced environmental footprints and broad application potential [1,2,3].

Natural fiber-reinforced biocomposites have emerged as one of the most promising classes of sustainable engineering materials because they combine environmental compatibility with desirable mechanical performance. Compared with conventional synthetic reinforcements, natural fibers offer several advantages, including low density, high specific strength and stiffness, renewability, biodegradability, widespread availability, and reduced carbon emissions throughout their life cycle. A wide range of lignocellulosic resources, including flax, hemp, jute, sisal, bamboo, coconut, banana, rice straw, and corn stalks, have been successfully incorporated into polymer matrices to improve the mechanical, thermal, and physical performance of composite materials. Beyond conventional natural fibers, increasing attention has been directed toward the valorization of agricultural residues as sustainable reinforcing agents. Converting these abundant lignocellulosic wastes into functional composite constituents not only minimizes environmental pollution and disposal challenges but also promotes resource efficiency by transforming low-value biomass into high-value engineering materials. The integration of agricultural waste into polymer composites represents an effective strategy for supporting circular economy principles while enabling the development of cost-effective and environmentally sustainable advanced materials [4,5,6].

Artichoke (*Cynara cardunculus* L.) is one of the most extensively cultivated vegetable crops in Mediterranean countries, generating substantial quantities of lignocellulosic residues, particularly stalks and leaves, during harvesting and processing. These agricultural by-products are generally discarded, composted, or utilized in low-value applications despite representing a valuable renewable biomass resource. Owing to their relatively high cellulose content, low density, and favorable fiber morphology, artichoke stalks have considerable potential as sustainable reinforcing materials for polymer composites. Their lignocellulosic structure promotes efficient stress transfer at the fiber–matrix interface and can enhance the stiffness and dimensional stability of biocomposites when appropriate processing and interfacial compatibility are achieved. Recent studies have demonstrated that artichoke-derived fibers can improve the mechanical performance of bio-based composites, particularly when incorporated into biodegradable polymer matrices. Nevertheless, the application of ground WAS as a reinforcement in multifunctional hybrid biocomposites, especially in combination with renewable bio-based modifiers, remains insufficiently explored. This research gap highlights the need for further investigations aimed at maximizing the value of artichoke agricultural residues while advancing the development of high-performance and environmentally sustainable polymer composites [7,8,9].

A major challenge associated with natural fiber-reinforced polymer composites is the inherently weak interfacial compatibility between hydrophilic lignocellulosic fibers and hydrophobic polymer matrices, which often results in poor stress transfer, reduced interfacial adhesion, and deterioration of the mechanical performance. To overcome this limitation, considerable research has focused on the incorporation of bio-based plasticizers, compatibilizers, and reactive modifiers capable of enhancing fiber–matrix interactions. Among these, MSO has attracted significant attention as a renewable and environmentally benign additive because of its low toxicity, biodegradability, and excellent compatibility with various polymer systems. In addition to improving the sustainability of composite formulations, modified soybean oil has been reported to increase polymer chain flexibility, reduce brittleness, enhance impact resistance, and promote stronger interfacial bonding between natural fibers and polymer matrices. These beneficial effects can contribute to improved mechanical durability and processing characteristics of biocomposites. Despite these advantages, studies investigating the synergistic use of MSO together with waste artichoke stem as reinforcing and functional components in sustainable biocomposite systems remain scarce, highlighting an important research opportunity for the development of high-performance green composite materials [10,11,12].

Polyurethane has emerged as an attractive polymer matrix for sustainable biocomposite development because its chemical structure, crosslinking density, and macroscopic properties can be tailored over a broad range through appropriate selection of polyols, isocyanates, and bio-based modifiers. In particular, polyester polyol-based polyurethanes reacted with methylenediphenyl diisocyanate provide a reactive network in which hydroxyl-functionalized renewable components can be incorporated during the polymer-forming reaction. This chemical versatility is particularly relevant for the utilization of modified vegetable oils, since chemical modification of vegetable oil can introduce hydroxyl functionalities that facilitate its integration into the polyurethane network and potentially influence chain mobility, crosslinking behavior, and the resulting mechanical, thermal, dielectric, and physical properties [13,14].

The performance of polymer composites is governed by the complex interaction of multiple formulation and processing parameters, including reinforcement loading, particle size, additive concentration, curing conditions, processing temperature, pressure, and fabrication time. These interacting variables give rise to highly nonlinear relationships that are often difficult to describe accurately using conventional statistical or empirical modeling approaches. In recent years, artificial intelligence (AI)-based techniques have emerged as powerful alternatives for modeling such complex material systems owing to their ability to capture nonlinear behavior without requiring explicit mathematical formulations. Among these techniques, ANNs have gained widespread recognition in materials science and engineering because of their capability to learn intricate input–output relationships directly from experimental datasets, deliver high predictive accuracy, and exhibit strong generalization performance. ANN models have been successfully applied to predict a wide range of composite properties, enabling efficient material design, process optimization, and significant reductions in experimental time and cost [15,16].

Although extensive research has been conducted on natural fiber-reinforced polymer composites, bio-based vegetable oil modifiers, and ANN applications in materials engineering, studies integrating these research areas into a unified framework remain limited. In particular, the combined utilization of WAS as a lignocellulosic reinforcement and MSO as a renewable functional additive has received little attention, especially when coupled with comprehensive machine learning (ML) approaches for simultaneous prediction of multiple material properties. Furthermore, comparative evaluations of different ANN training algorithms for modeling the electrical, thermal, physical, and mechanical behavior of such sustainable biocomposites are rarely reported. This lack of integrated studies highlights a significant research gap in both the optimization of environmentally friendly composite materials and the application of artificial intelligence as a reliable decision-support tool for advanced material design and property prediction [17,18,19,20].

In this study, PUBs incorporating WAS as a lignocellulosic reinforcement and MSO as a bio-based formulation modifier are developed and systematically evaluated. A total of 48 formulations are prepared by varying the WAS content from 0 to 3.5 wt.% and the MSO content from 0 to 5 wt.%, and their dielectric constant, thermal conductivity, Shore A hardness, bulk density, tensile strength, and elongation at break are experimentally determined. To complement the experimental investigation, a multi-output ANN model is developed using WAS and MSO contents as input variables and the six measured properties as simultaneous outputs. Three training algorithms, namely LM, BR, and SCG, are comparatively assessed using 42 samples for model development and six independent samples for external validation. The main objective is to establish an efficient experimental–computational framework capable of describing the formulation–property relationships of WAS/MSO-modified PUBs and identifying a reliable ANN training strategy for simultaneous prediction of their multifunctional properties. The findings are expected to demonstrate the potential of combining agricultural-waste valorization, bio-based modification, and ANN-assisted prediction as a practical approach for reducing experimental effort and supporting the rational design and performance assessment of PUBs.

## 2. Materials and Methods

### 2.1. Materials

A commercial two-component polyurethane system comprising a polyester polyol (Part A) and 4,4′-methylenediphenyl diisocyanate (MDI, Part B) was used as the polymer matrix. The components were combined at the manufacturer-recommended 1:1 mixing ratio. The polyurethane network was formed through the reaction of the hydroxyl groups of the polyester polyol with the isocyanate groups of MDI, resulting in urethane linkages. A commercial cold-pressed, pure, and natural soybean oil (obtained from a local market in Ankara, Türkiye) was used as the precursor for the preparation of MSO. The soybean oil was subjected to sequential epoxidation and hydroxylation reactions. Hydrogen peroxide, acetic acid, and hydrochloric acid were used during epoxidation, whereas methanol, distilled water, and sulfuric acid were employed during hydroxylation. Hydrogen peroxide (30%), acetic acid (≥99.8%), and hydrochloric acid (37%), all purchased from Merck (Darmstadt, Germany), were used during the epoxidation step. Methanol (≥99.8%) and sulfuric acid (98%), also obtained from Merck (Darmstadt, Germany), were employed during the hydroxylation step. Distilled water was used throughout the chemical modification and purification procedures. All chemicals were used as received without further purification [21,22,23].

The modified oil was subsequently subjected to neutralization and purification before incorporation into the polyurethane formulations. WAS was employed as the lignocellulosic reinforcement. The collected artichoke stems were thoroughly washed to remove adhering impurities and dried at 45 ± 2 °C to constant mass. The dried stems were mechanically ground and sieved, and the fraction with a particle-size range of 77–149 μm was used in all formulations. The prepared WAS was stored in airtight containers under dry laboratory conditions until composite fabrication to minimize moisture uptake [21,22,23].

### 2.2. Preparation of WAS and MSO

The WAS was collected from local agricultural production areas following the harvesting season and used as a lignocellulosic reinforcement in the polyurethane biocomposites. The collected stems were thoroughly washed with tap water and subsequently rinsed with distilled water to remove adhering soil, dust, and other surface contaminants. The cleaned material was dried in a forced-air convection oven at 45 ± 2 °C until a constant mass was obtained. The dried stems were then mechanically ground and sieved, and the particle fraction in the range of 77–149 μm was selected for composite fabrication. The prepared WAS powder was stored in airtight containers under dry conditions at room temperature until use to minimize moisture uptake and maintain consistent material conditions prior to processing.

MSO was prepared through a sequential epoxidation–hydroxylation route. Initially, soybean oil was subjected to epoxidation using hydrogen peroxide, acetic acid, and hydrochloric acid. In this step, the carbon–carbon double bonds of the unsaturated fatty-acid chains were converted into oxirane groups. The resulting epoxidized soybean oil was subsequently subjected to hydroxylation using methanol and distilled water in the presence of sulfuric acid as the catalyst. This treatment converted the oxirane functionalities into hydroxyl-containing groups, thereby increasing the hydroxyl functionality of the modified oil. Following completion of the reaction sequence, the product was neutralized and purified to remove residual acidic species and reaction-derived impurities. The purified product was designated as MSO and subsequently incorporated into the polyurethane formulations [21,22].

In the present study, MSO was incorporated as a bio-based modifying component, whereas WAS served as the lignocellulosic reinforcement. The incorporation of MSO was associated with systematic changes in the measured physical, electrical, thermal, and mechanical properties of the polyurethane biocomposites. These changes can reasonably be attributed to the hydroxyl functionality introduced during soybean-oil modification, which provides additional polar groups that may interact with the hydroxyl-containing polyester polyol and the lignocellulosic constituents of WAS. The observed variations in the mechanical properties of the developed PUBs may be associated with changes in the WAS and MSO contents and their distribution within the polymer matrix. The incorporation of these components could alter the internal structure and local stress distribution of the composite, thereby influencing its tensile strength and hardness. In particular, variations in the dispersion and distribution of the incorporated phases may contribute to the changes observed in the mechanical response. At relatively high contents, localized accumulation or non-uniform distribution of the incorporated phases could also be associated with the observed changes in mechanical performance. The mechanical behavior of the PUBs appears to reflect the combined effects of the WAS and MSO contents and their distribution within the polymer matrix. The experimental results indicate that the combined adjustment of WAS and MSO contents provides an effective means of tailoring the density, Shore A hardness, tensile strength, elongation at break, dielectric constant, and thermal conductivity of the resulting PUBs. MSO acts as a bio-based formulation modifier that contributes to the tunability of the polyurethane composite system while WAS provides an agricultural-waste-derived lignocellulosic reinforcement [21,22,23].

### 2.3. Production of Sustainable PUBs

A total of 48 experimental formulations were designed to systematically investigate the combined effects of WAS and MSO on the properties of the developed PUBs (Table 1). The WAS content was varied from 0.0 to 3.5 wt.% at 0.5 wt.% intervals, while the MSO content was adjusted from 0 to 5 wt.% at 1 wt.% increments, resulting in a full factorial experimental design covering all possible combinations of the selected composition ranges. This experimental matrix enabled a comprehensive evaluation of the individual and interactive influences of both variables on the electrical, thermal, physical, and mechanical performance of the biocomposites. The generated dataset also provided a balanced and representative input matrix for the development, training, validation, and testing of the ANN models, thereby ensuring reliable prediction and comparative assessment of the investigated material properties [23,24].

Following homogeneous mixing, the composite mixture was transferred into silicone molds treated with a mold-release agent to facilitate specimen removal after curing. To improve structural integrity and reduce the formation of internal defects, the filled molds were placed under vacuum for approximately 15 min, allowing trapped air bubbles to be effectively removed. Subsequently, the samples were left to cure under controlled laboratory conditions at a temperature of 40 ± 1 °C for 24 h. Upon completion of the curing process, all samples were conditioned under standard laboratory conditions for 24 h before undergoing electrical, thermal, physical, and mechanical characterization to ensure uniform testing conditions and reproducible experimental results.

PUBs are prepared using 4,4′-MDI, polyester polyol, MSO, and WAS as the main components. The WAS content is varied from 0.0 to 3.5 wt.%, while the MSO content is varied from 0 to 5 wt.%, resulting in a total of 48 different formulations. All composition values are calculated based on the total mass of the final formulation.

For the preparation of the composite formulations, the predetermined amount of WAS is initially incorporated into the polyester polyol. The polyester polyol–WAS mixture is mechanically mixed at 900 rpm for 15 min to promote homogeneous dispersion of the WAS particles throughout the polyol phase. The predetermined amount of MSO is then incorporated into the formulation and mixed to obtain a homogeneous precursor mixture. The prepared mixture was subsequently sonicated for approximately 3 min using a SCIENTZ ultrasonic homogenizer (JY92-IIN, Ningbo Scientz Biotechnology Co., Ltd., Zhejiang, China) equipped with a 6 mm titanium probe, operating at a frequency of 20 kHz and a maximum power specification of 650 W, to facilitate homogenization and uniform dispersion of the components. Subsequently, 4,4′-MDI is added to the prepared polyol-based mixture, and the resulting system is mixed at 1500 rpm for 1 min. This relatively short mixing period after MDI addition is used to ensure effective homogenization while limiting excessive reaction and viscosity increase during processing.

Immediately after mixing, the reactive polyurethane mixture is transferred into the appropriate molds by casting. The molded specimens are then cured at 40 °C for 24 h to promote the formation of the polyurethane network through the reaction between the isocyanate (–NCO) groups of 4,4′-MDI and the hydroxyl (–OH) groups of the polyester polyol and MSO. After completion of the curing process, the specimens are removed from the molds and conditioned prior to characterization.

**Figure 1:**
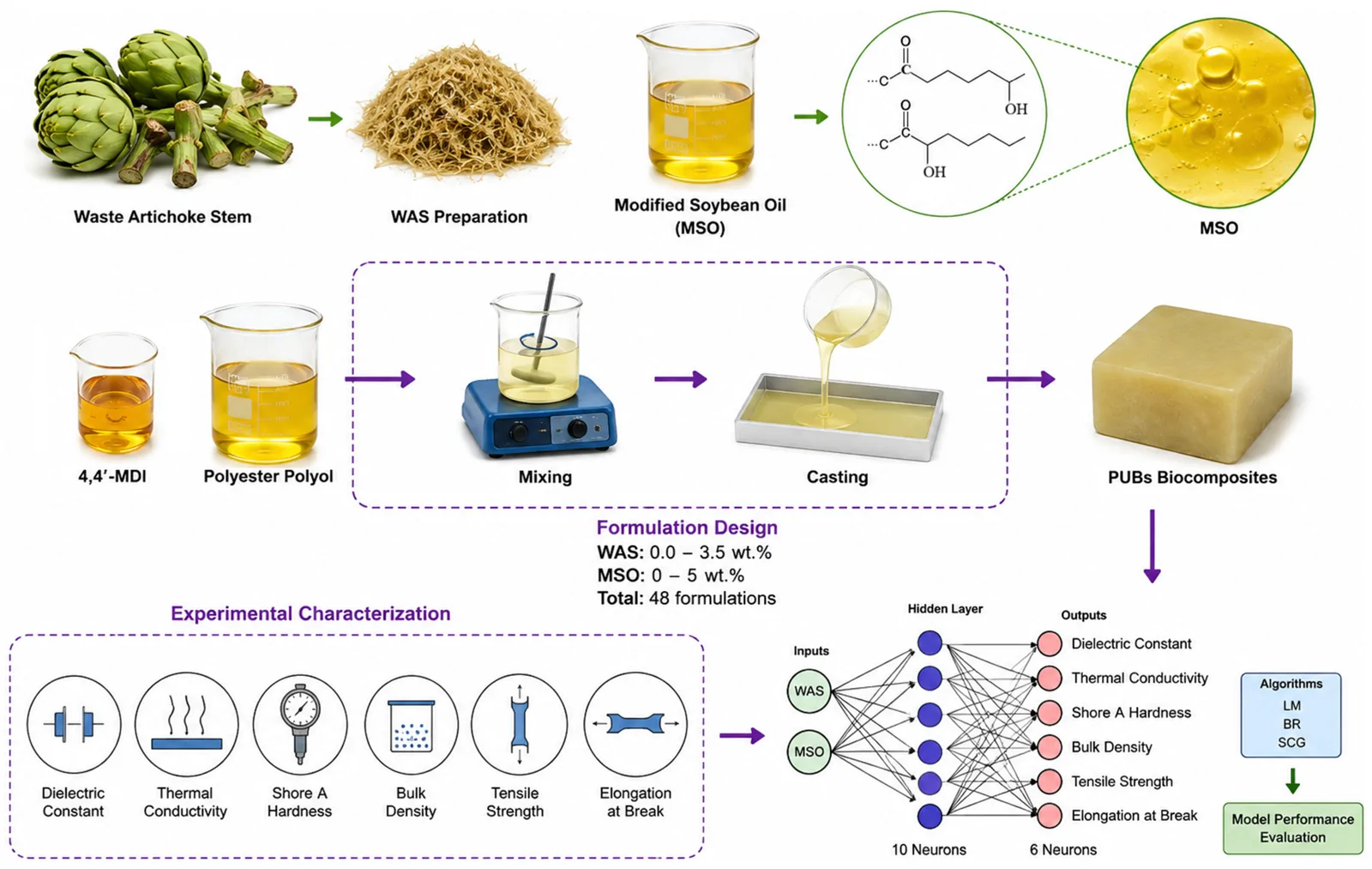
Schematic illustration of the production procedure and experimental workflow for PUBs.

Figure 1 presents the experimental workflow for the fabrication of PUBs. Initially, WAS was collected, washed, dried, ground, and sieved to obtain a homogeneous lignocellulosic filler. The matrix was then prepared by mixing polyester polyol and 4,4′-MDI at the prescribed ratio, followed by the incorporation of MSO as a renewable bio-based modifier. The prepared constituents were homogenized through mechanical mixing, and vacuum degassing before being cast into silicone molds and cured under controlled conditions to produce the composite specimens. A total of 48 SEB formulations were fabricated by systematically varying the WAS and MSO contents according to the experimental design. Finally, the produced PUBs were characterized in terms of their electrical, thermal, physical, and mechanical properties, and the resulting experimental dataset was used to develop and comparatively evaluate the ANN models employing different training algorithms.

### 2.4. Bulk Density Measurement

The bulk density of the produced PUBs was measured in accordance with ASTM D792 method [25]. For each formulation, the specimens were first weighed in air and subsequently immersed in distilled water to determine their apparent mass under submerged conditions. The bulk density values were calculated from the measured mass difference by applying the principle and considering the density of the immersion medium. To ensure the accuracy and repeatability of the measurements, a minimum of three specimens from each biocomposite formulation were analyzed, and the reported density values represent the arithmetic mean of the individual measurements [26].

### 2.5. Shore A Hardness Test

The surface hardness of the produced polyurethane-based biocomposites was evaluated in accordance with ASTM D2240-15 using an LX-D-2 Shore A durometer (HUATEC Group Corporation, Beijing, China). Hardness measurements were performed at three randomly selected positions on each specimen to minimize the influence of local surface variations and material heterogeneity. The durometer was applied to the specimen surface under identical testing conditions, and the indentation resistance recorded at each measurement position was used to characterize the hardness of the composite structure. For each formulation, three independent hardness readings were obtained, and the arithmetic mean of these three readings was calculated and reported as the Shore A hardness value. All measurements were performed under the same testing conditions to ensure consistency and repeatability among the different formulations. The use of three measurements per formulation provided an averaged value that reduced the potential influence of localized variations in the composite surface and improved the reliability of the reported hardness results [27,28].

### 2.6. Tensile Test

The mechanical behavior of the fabricated biocomposites was evaluated through tensile testing in accordance with ASTM D638 [29]. Dumbbell-shaped specimens conforming to the geometry were prepared using silicone molds, with a total specimen length of 115 mm, a gauge length of 25 mm, a narrow-section width of 6 mm, and a narrow-section length of 33 mm. The tensile tests were performed using a UTEST universal testing machine (Ankara, Türkiye) equipped with a 1 kN load cell. The tests were conducted at a constant crosshead speed of 50 mm min^−1^ under identical laboratory conditions [30].

### 2.7. Dielectric Constant

The dielectric behavior of the developed PUBs was characterized using a Fytronix precision LCR meter (Fytronix, Elazığ, Türkiye) in accordance with ASTM D150. Dielectric measurements are performed at a fixed frequency of 1 kHz using circular specimens with uniform dimensions under identical measurement conditions. For each formulation, the dielectric constant is measured in three repeated measurements, and the arithmetic mean value is used as the representative experimental result. The 1 kHz dielectric constant values are directly used as the dielectric-property output in the ANN model. The experimental results were subsequently analyzed to assess the effects of WAS and MSO contents on the dielectric performance of the produced biocomposites [31,32].

### 2.8. Thermal Conductivity Analysis

The thermal conductivity of the produced PUBs was determined using a TLS-100 steady-state thermal conductivity analyzer (Thermtest Inc., Fredericton, NB, Canada). Prior to the measurements, all specimens were conditioned under standard laboratory conditions to minimize the influence of environmental factors and to ensure consistency among the measurements. The thermal conductivity measurements were performed under steady-state heat transfer conditions in accordance with the operating procedure of the instrument. For each formulation, three independent specimens were tested under identical experimental conditions, and a separate thermal conductivity measurement was obtained for each specimen. The three measured values were then averaged, and the resulting arithmetic mean was reported as the thermal conductivity value of the corresponding formulation. The use of three independent specimens for each formulation was intended to account for possible specimen-to-specimen variations and to improve the reliability and repeatability of the experimental results. The averaged thermal conductivity values were subsequently used to evaluate the effects of WAS and MSO contents on the heat-transfer characteristics of the developed biocomposites [33,34].

### 2.9. ANN Modeling

In this study, WAS and MSO are incorporated into the polyurethane matrix at systematically varied concentrations to develop PUBs. WAS is employed as a lignocellulosic reinforcement derived from an agricultural waste stream, while MSO is incorporated as a bio-based modifier. The WAS content is varied from 0 to 3.5 wt.% at 0.5 wt.% intervals, whereas the MSO content is varied from 0 to 5 wt.% at 1 wt.% intervals. This experimental design results in 48 distinct WAS–MSO formulations, providing a systematic dataset for evaluating the effects of formulation variables on the resulting material properties. The WAS and MSO contents are subsequently defined as the two input variables of the ANN model.

The experimental performance of each formulation is evaluated in terms of six response variables, namely dielectric constant, thermal conductivity, Shore A hardness, bulk density, tensile strength, and elongation at break. These properties collectively represent the electrical, thermal, physical, and mechanical characteristics of the developed PUBs. The experimentally obtained values are used as target outputs for ANN modeling. Accordingly, the ANN establishes a nonlinear relationship between the two formulation parameters (WAS and MSO contents) and the six experimentally determined material properties. This multi-output modeling approach enables the simultaneous prediction of the investigated properties from the formulation composition and provides a computationally efficient framework for evaluating the influence of WAS and MSO contents without requiring an independent predictive model for each response [35,36].

### 2.10. ANN Training Algorithms

The quest to reduce the environmental impact of industrial waste has made it necessary to utilize agricultural fibers in the production of biocomposites, thereby creating a sustainable alternative in materials engineering. However, the structural complexity and inter-component variability of natural fibers present a series of production challenges that make it difficult to predict mechanical properties using traditional experimental methods. In this context, ANNs demonstrate superior capability in minimizing the costs of experimental trial and error by modeling the complex and nonlinear nature of fiber-matrix interactions [35,36]. This neural network study aims to optimize the performance of biocomposites and strengthen decision-support mechanisms in production processes by systematically analyzing the effectiveness of various training algorithms on this prediction process. This modeling approach, which specifically utilizes the LM, BR, and SCG algorithms, aims to minimize error margins by directly influencing training performance and generalization capacity [37,38,39,40].

The optimization capabilities of these algorithms during the training process enhance the model’s reliability by enabling the attainment of high correlation coefficients across different composite structures [41]. In particular, the LM algorithm’s fast convergence and high accuracy make it one of the most widely preferred approaches in the literature for modeling complex mechanical relationships [42]. In contrast, while the BR algorithm offers more stable generalization performance by preventing the problem of overfitting, the SCG approach provides effective optimization on large datasets with low computational cost [43,44]. In this context, the predictive power of the different algorithms under investigation enables the mapping of nonlinear dependencies among complex material variables with high accuracy [45]. Thus, a computational approach that rapidly optimizes material design parameters and has proven its reliability is replacing the time-consuming and costly nature of traditional experimental processes [46,47].

To develop the ANN model, the data obtained from experimental studies were imported into the MATLAB R2015a. The dataset consists of a total of 48 observations. To accurately assess the model’s generalization ability, the dataset was divided into two groups: training data and independent test data. A total of 42 data points were used to train the model, while the remaining 6 data points were set aside as independent test data—excluded from model training—solely for the purpose of performance evaluation. In this way, the prediction performance of the developed model on examples it had not previously seen was objectively evaluated.

**Figure 2:**
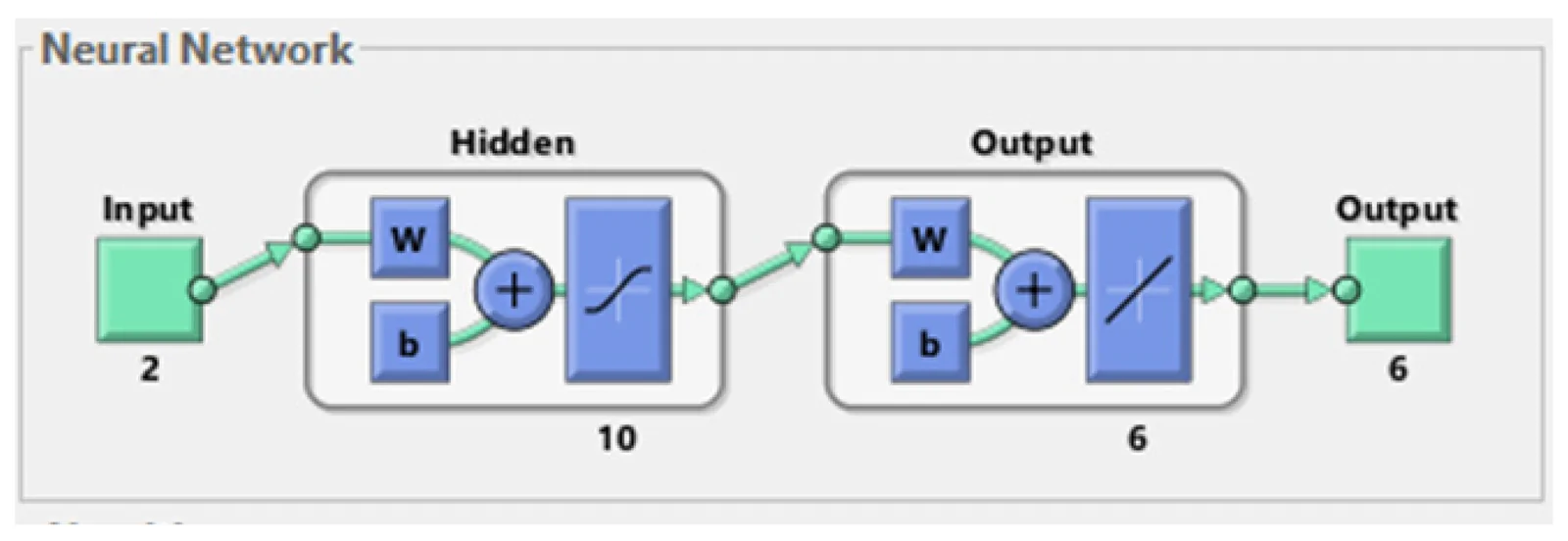
Schematic representation of the multi-output ANN architecture used for simultaneous prediction of six material properties.

The MATLAB Neural Network Toolbox was used to model the experimental data. The developed model is a feedforward multilayer ANN. Using the experimental data, a multi-output ANN model consisting of two inputs and six outputs was developed, as shown in Figure 2. The network architecture used 10 hidden neurons, and the LM, BR, and SCG training algorithms were compared. The same 42 data points from the data presented in Table 1 were used in training the network structure using these three training algorithms. Data points 5, 10, 19, 28, 38, and 47 from Table 1 were separated as external control data and compared by applying them to the ANN structure with three different training algorithms.

The input variables used were the percentage of WAS and the percentage of MSO. The output variables consist of, in order, the dielectric constant, thermal conductivity, Shore A hardness, bulk density, tensile strength, and elongation at break. Thus, six different material properties were predicted simultaneously using a single artificial neural network model.

### 2.11. LM Training Algorithm

The LM algorithm is one of the second-order optimization methods and is known for its high convergence rate, particularly for small and medium-sized datasets. By combining the gradient descent and Gauss–Newton methods, the algorithm rapidly reaches the minimum of the error function. Due to its fast convergence, it is widely used in engineering applications [48].

### 2.12. BR Training Algorithm

The BR algorithm reduces overfitting by regularizing the network weights during training. Thanks to this feature, it achieves high generalization performance, particularly in problems with a limited amount of experimental data. Furthermore, because it produces more reliable predictions on independent test data, it is frequently used in materials modeling studies [49].

### 2.13. SCG Training Algorithm

The SCG algorithm is one of the iterative optimization methods that operates without the need to compute second derivatives. Although its computational cost is low, its convergence rate and prediction accuracy may vary depending on the structure of the dataset. While it offers advantages, particularly with large datasets, it may perform worse than the LM and BR algorithms with small datasets [50].

## 3. Results and Discussion

### 3.1. LM Training Algorithm Performance Results

The multi-output artificial neural network model developed in this study was first trained using the LM training algorithm. The LM algorithm was chosen because it is a method based on a second-order optimization approach that provides rapid convergence, particularly for small- to medium-sized datasets. In the model, two input variables (WAS and MSO ratios) and six output variables (dielectric constant, thermal conductivity coefficient, Shore A hardness, bulk density, tensile strength, and elongation at break) were estimated simultaneously.

**Figure 3:**
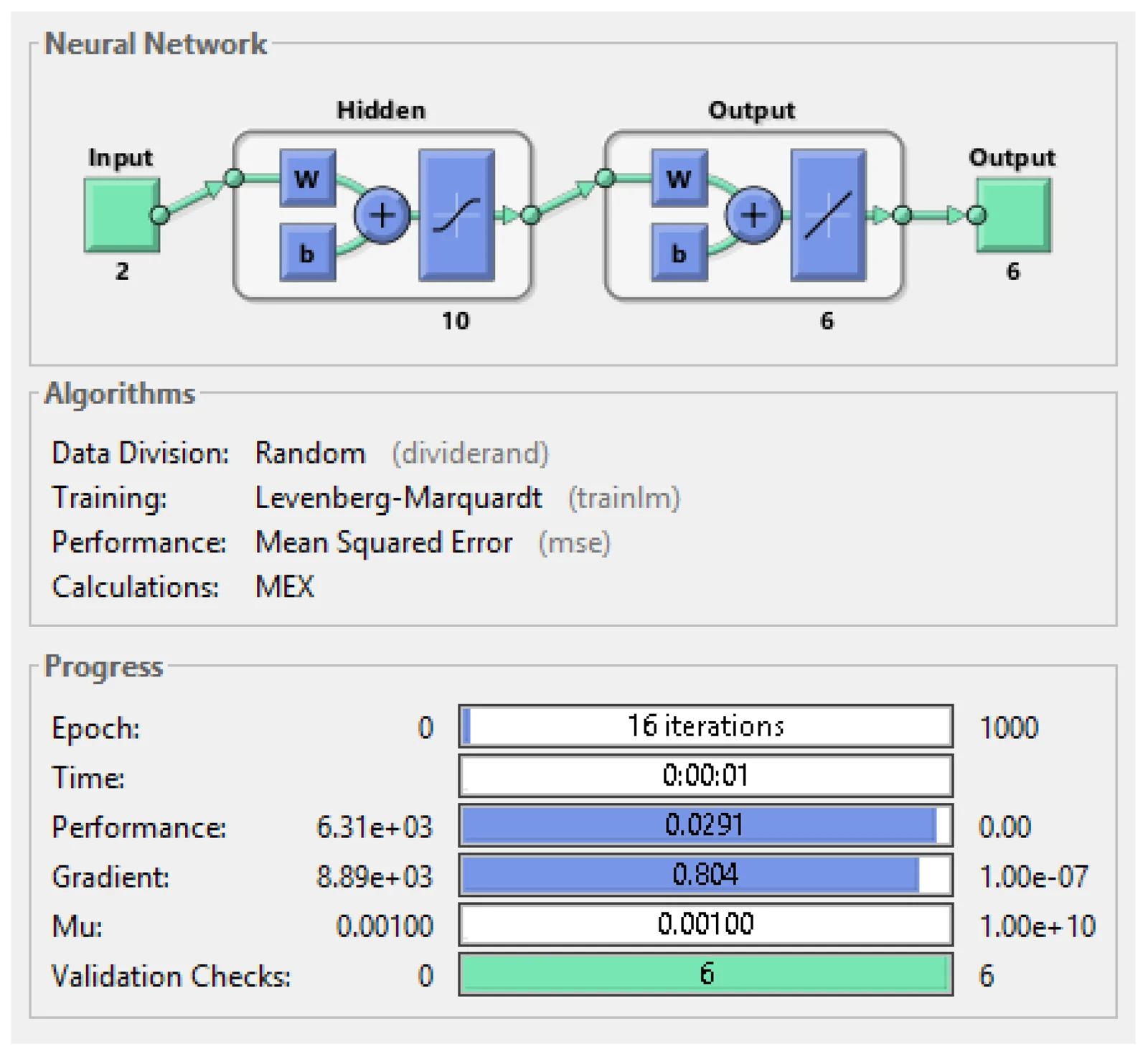
LM training algorithm: ANN architecture and results.

The performance of the developed ANN for selected data was initially evaluated using the LM training algorithm. The experimental dataset consisted of 42 observations, which were randomly divided into three subsets comprising 30 samples for network training, 6 samples for validation, and 6 samples for independent testing. The training subset was employed to optimize the network weights and biases, whereas the validation subset was used to monitor the learning process and prevent overfitting through the early stopping criterion. The independent test subset, which was excluded from the training process, served to assess the generalization capability of the developed model. Model performance was comprehensively evaluated using multiple statistical and graphical criteria, including mean squared error (MSE), root mean square error (RMSE), mean absolute error (MAE), mean absolute percentage error (MAPE), correlation coefficient (R), learning performance curves, error histogram analysis, regression analysis, and training parameters [51]. The obtained results demonstrated that the LM algorithm successfully learned the nonlinear relationships between the input variables and the six output properties, providing high prediction accuracy and reliable generalization performance for the developed multi-output ANN model for selected data. In addition, Figure 3 shows the network architecture used with the LM algorithm and the performance results.

**Figure 4:**
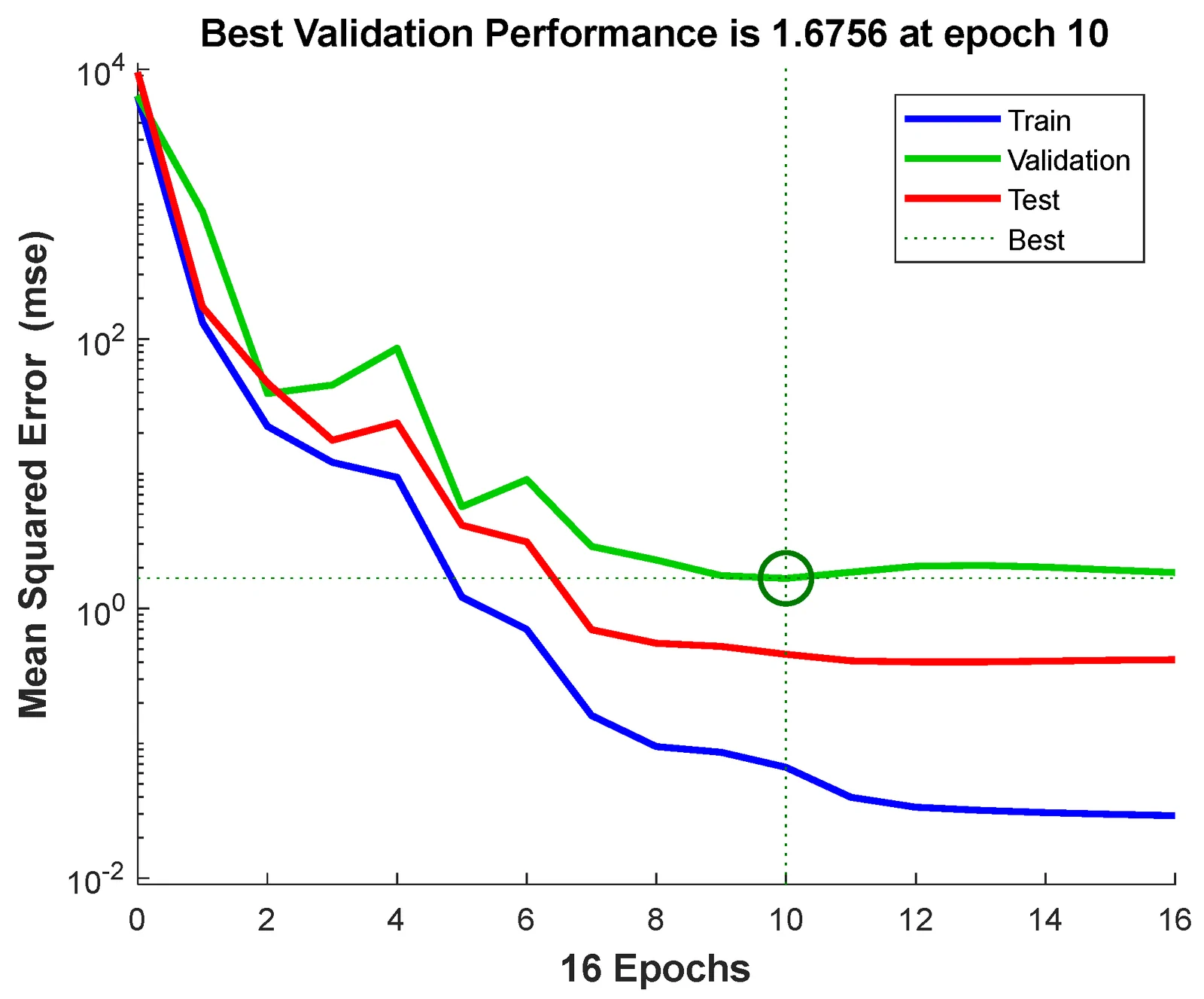
Performance curves of the LM training algorithm.

When examining the training performance of the LM algorithm in Figure 4, it can be seen that the MSE value decreases steadily throughout the training process. The lowest error value for the validation set was obtained at the 10th epoch, at 1.6756; training was terminated at the 16th epoch by applying the early stopping criterion, as the validation performance failed to improve over six consecutive epochs. This helped preserve the model’s generalization ability by preventing it from overfitting.

The fact that the training and validation curves shown in Figure 4 run very close to each other indicates that the model did not merely memorize the training data and that it demonstrates similar performance across different data sets. The steady decline in the error curve during the training process indicates that the optimization algorithm is converging steadily and that the network parameters are being updated appropriately. These results demonstrate that the LM algorithm successfully completed the learning process on the dataset used [52].

**Figure 5:**
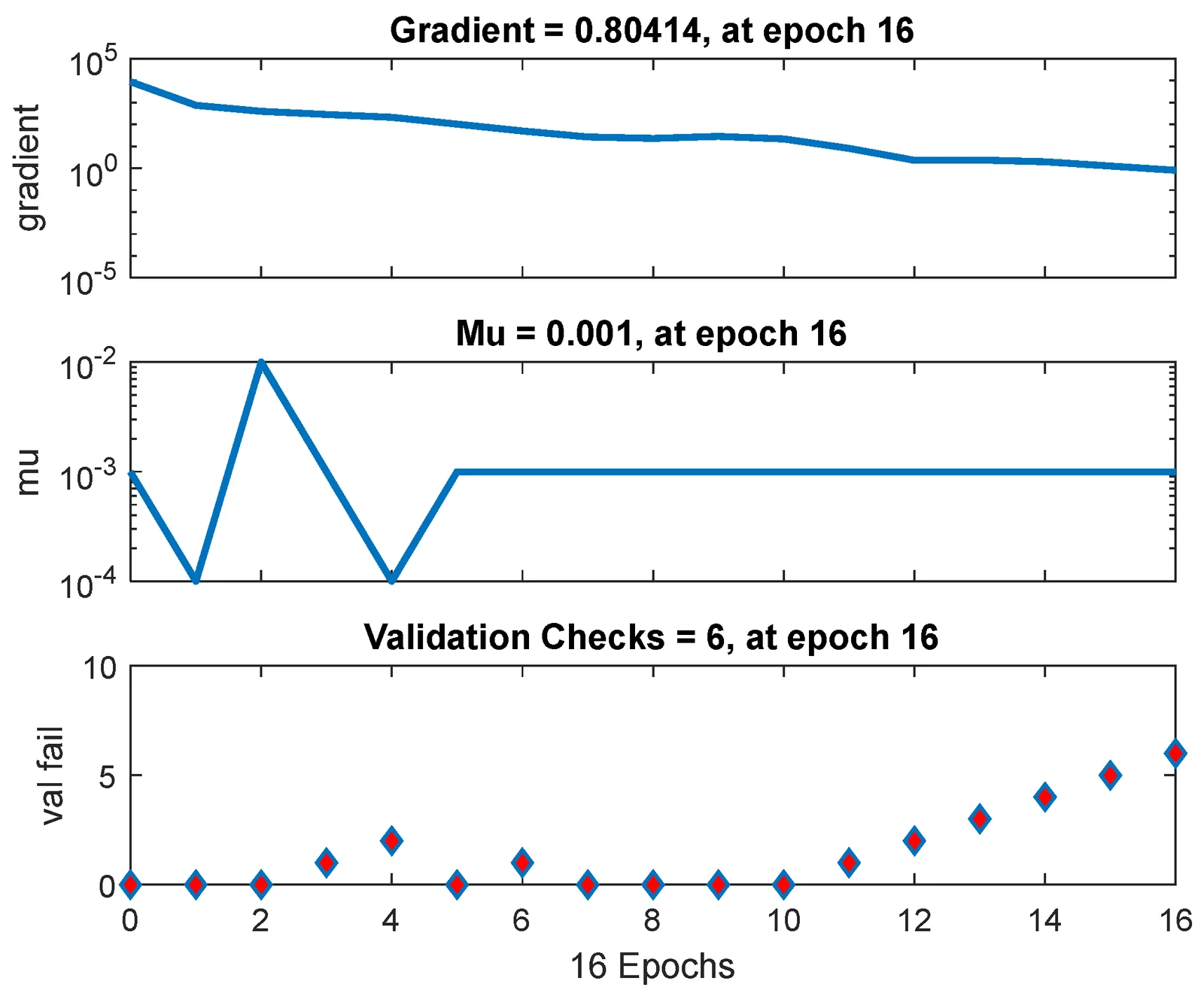
Graphs of gradient, Mu, and validation checks for the LM training algorithm.

During the training process of the LM algorithm, the gradient value shown in Figure 5 steadily decreased as the epochs progressed and was calculated as 0.80414 in the final epoch. This decrease in the gradient value indicates that a steady convergence toward the minimum point of the loss function has occurred. Additionally, the damping coefficient (μ) specific to the LM algorithm reached a value of 0.001 at the end of training. This indicates that while the algorithm initially exhibited gradient descent behavior, it transitioned to the Gauss–Newton method in later stages, thereby achieving faster and more stable optimization.

The fact that training is automatically halted when the validation check value shown in Figure 5 reaches 6 does not mean that the model has made a mistake. On the contrary, this mechanism prevents overfitting by terminating training when there is no further improvement in validation performance. Thus, the network architecture obtained at the end of training is preserved in a way that allows it to make successful predictions not only on the training data but also on independent samples.

**Figure 6:**
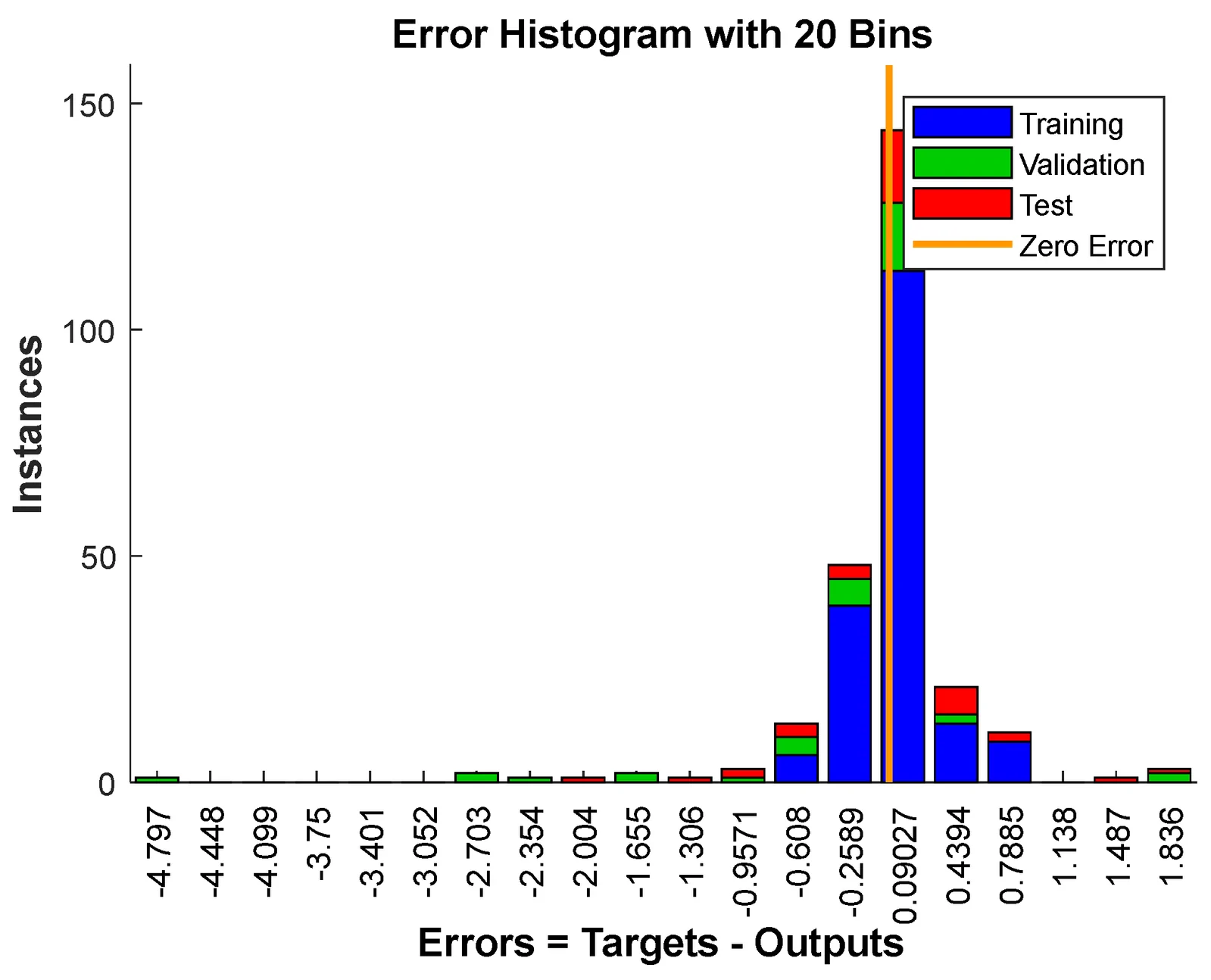
LM training algorithm error histogram.

An examination of the error histogram for the LM algorithm, as shown in Figure 6, reveals that the majority of prediction errors are concentrated around zero. The symmetrical nature of the error distribution indicates that there is no systematic bias in the model and that the predictions are evenly distributed in both the positive and negative directions. Furthermore, the fact that there are very few samples in wide error intervals demonstrates that the model produces estimates that are quite close to the experimental values for most samples. The narrow distribution of the error histogram is one of the key indicators of high model accuracy. In particular, the fact that similar error behavior is observed for all output variables in multi-output problems supports the reliability of the developed network architecture [53].

**Figure 7:**
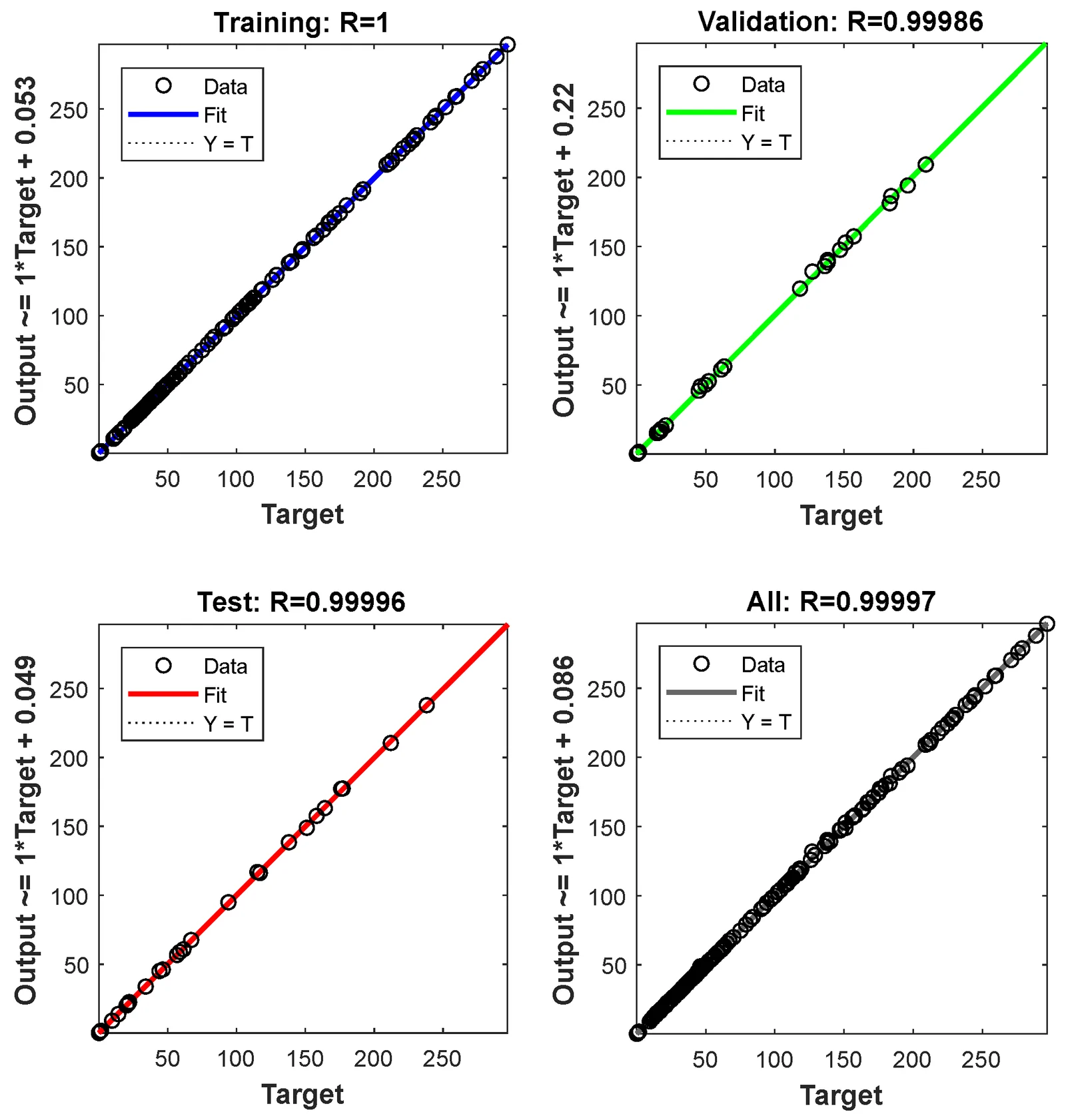
Regression results of the LM training algorithm. In the regression equations, the asterisk (*) symbol denotes the multiplication operator.

One of the most important metrics used in evaluating model performance is the linear relationship between experimental values and artificial neural network predictions. As shown in Figure 7, the correlation coefficients for the training, validation, test, and entire datasets in the regression analysis were calculated as 1.0000, 0.99986, 0.99996, and 0.99997, respectively, using the LM algorithm. These values indicate that the predicted results almost perfectly align with the experimental measurements. The fact that the regression lines largely coincide with the ideal line indicates that there is no significant deviation in the model. Furthermore, the fact that the correlation coefficients for the training, validation, and test datasets are very close to one another shows that the model not only fits the training data but can also make predictions with high accuracy on independent samples in dataset.

When the results are evaluated collectively, it is evident that the LM algorithm achieved highly successful learning on the biocomposite dataset used. Thanks to its low validation error, high correlation coefficients, narrow error distribution, and early stopping mechanism, the model has demonstrated both high accuracy and strong generalization ability. In particular, the LM algorithm’s fast convergence property in small datasets has reduced training time while also improving prediction accuracy.

### 3.2. BR Training Algorithm Performance Results

The multi-output ANN model developed in this study was trained using the BR training algorithm. The developed multi-output artificial neural network was subsequently trained using the BR algorithm to evaluate its predictive capability for the experimental biocomposite dataset. A total of 42 experimental observations in the dataset were used in the network architecture to train the ANN, with the BR training algorithm selected. The MATLAB Neural Net Fitting Toolbox randomly selected 36 data points for training the BR method, using 30 of them for network training and the remaining 6 for testing the network. The BR training algorithm does not perform validation by default in the Neural Net Fitting Toolbox. The user has no option to intervene in this process. Unlike conventional training approaches, the BR algorithm incorporates a regularization mechanism that automatically controls the complexity of the network by optimizing both the prediction error and the network weights during training, thereby eliminating the need for a separate validation phase. Model performance was assessed using the MSE, RMSE, MAE, MAPE, R, regression analysis, error histogram, performance curves, and independent test results. The obtained training statistics indicated an extremely low prediction error together with a correlation coefficient approaching unity, demonstrating that the developed ANN for dataset successfully captured the nonlinear relationships between the formulation variables and the multiple output responses. These findings confirm that the BR algorithm provided excellent prediction accuracy, robust generalization capability, and reliable performance for modeling the electrical, thermal, physical, and mechanical properties of the developed PUBs [54].

**Figure 8:**
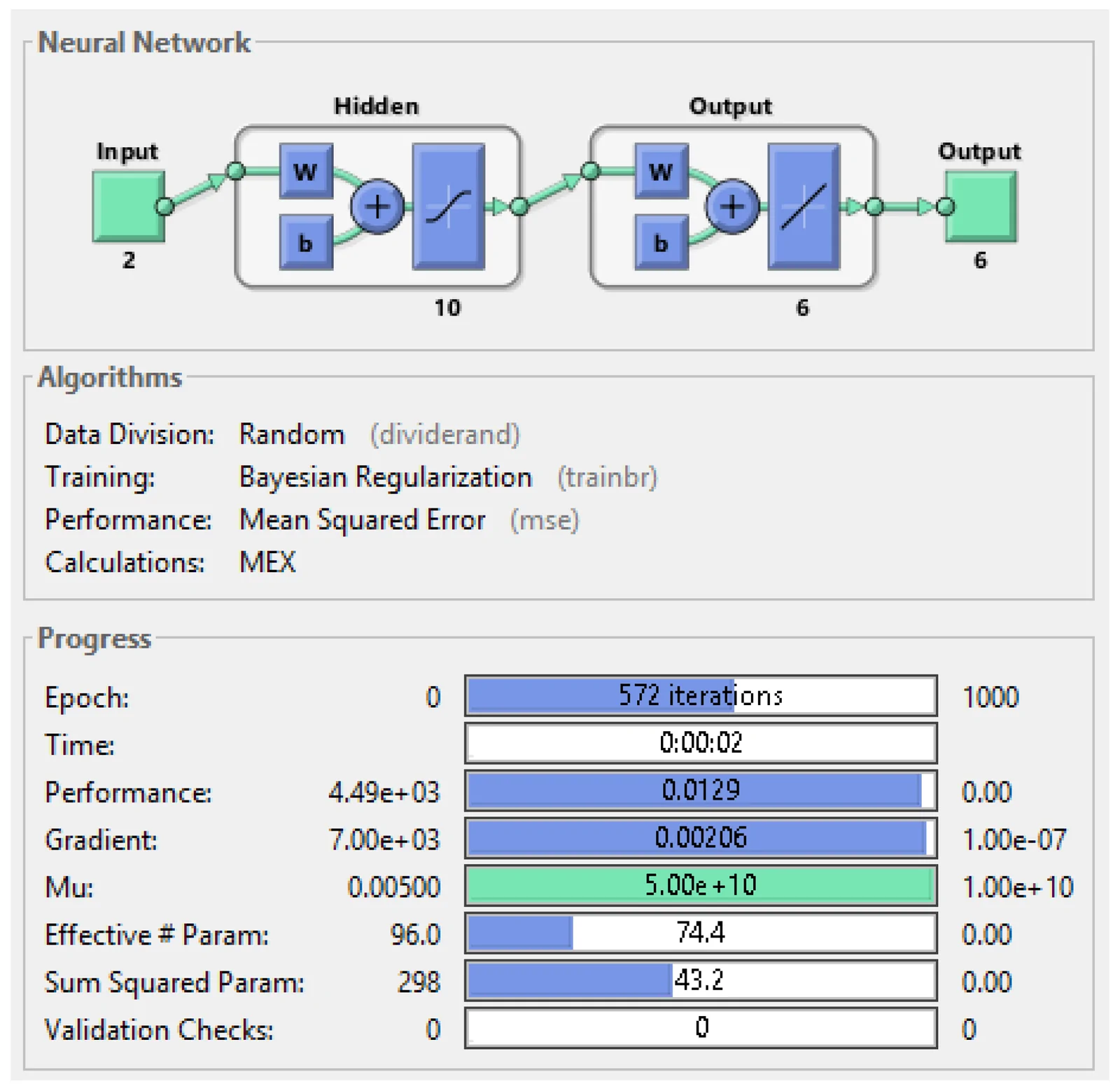
BR training algorithm: ANN architecture and results.

BR is an advanced training algorithm that controls model complexity by regularizing network weights during training and, in particular, reduces the risk of overfitting when dealing with a limited amount of experimental data. For this reason, it is widely preferred in engineering applications—such as those involving PUBs—where obtaining experimental data is time-consuming and costly. The performance of the BR algorithm was evaluated by considering the training process, error distribution, regression analysis, and independent test results. The network architecture selected for the application and the performance results of the BR algorithm are presented in Figure 8.

**Figure 9:**
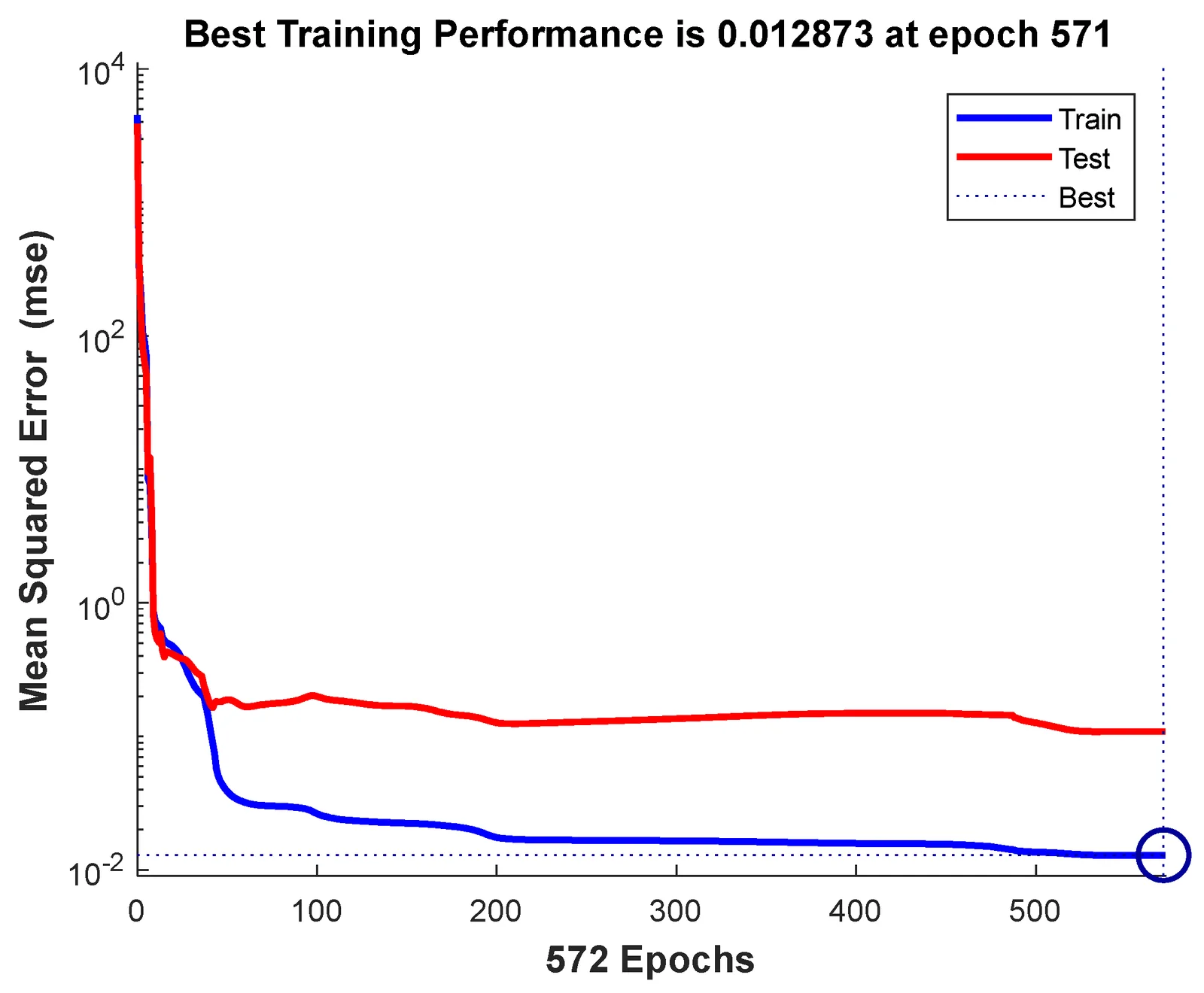
Performance curves of the BR training algorithm.

Figure 9 shows that, during the training process carried out using the BR training algorithm, the error values decreased steadily and the model exhibited stable convergence behavior. As can be seen from the performance curves in Figure 9, the performance curves progressed steadily throughout training. This indicates that the optimization process was stable and that the network successfully learned the nonlinear relationships among the experimental data.

**Figure 10:**
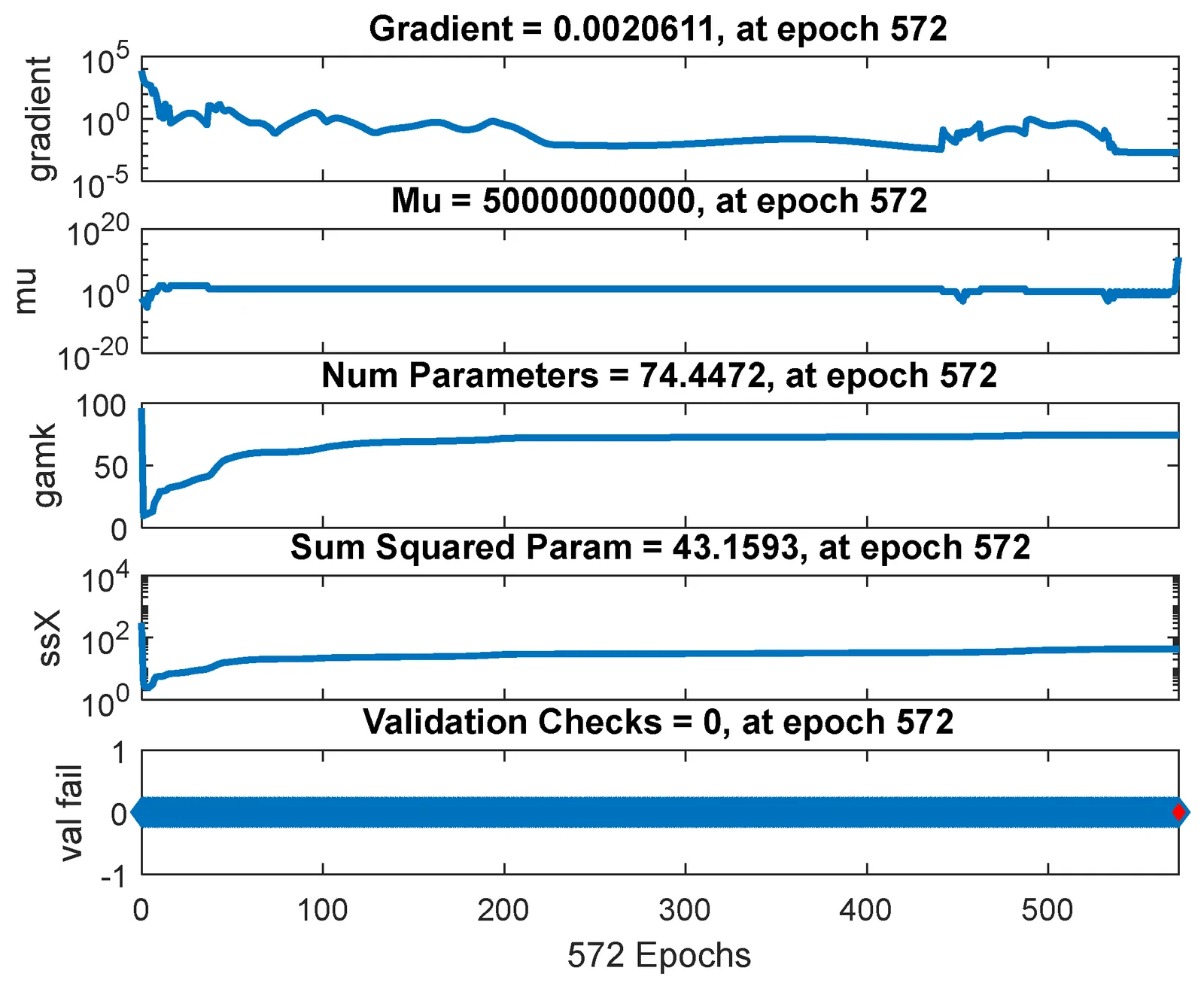
BR training algorithm: plots of gradient, Mu, and validation checks.

During the training process of the BR algorithm, it was observed that the gradient value shown in Figure 10 fluctuated around zero as the epochs progressed and reached a value of 0.0020611 at the 572nd epoch. Additionally, the damping coefficient (mu) specific to the BR algorithm reached a value of 50,000,000,000 at the end of training. As a result, the number of iterations increased, leading to slower performance.

One of the most significant advantages of the BR algorithm is that it regularizes network parameters without requiring a classical validation-based early stopping method. Thus, during training, it not only reduces the error but also controls model complexity, resulting in stronger generalization ability. This approach ensures more reliable predictions, particularly in limited datasets such as the one used in this study. The BR algorithm produced a stable solution at the end of training and did not exhibit a tendency toward overfitting during the training process for selected dataset.

**Figure 11:**
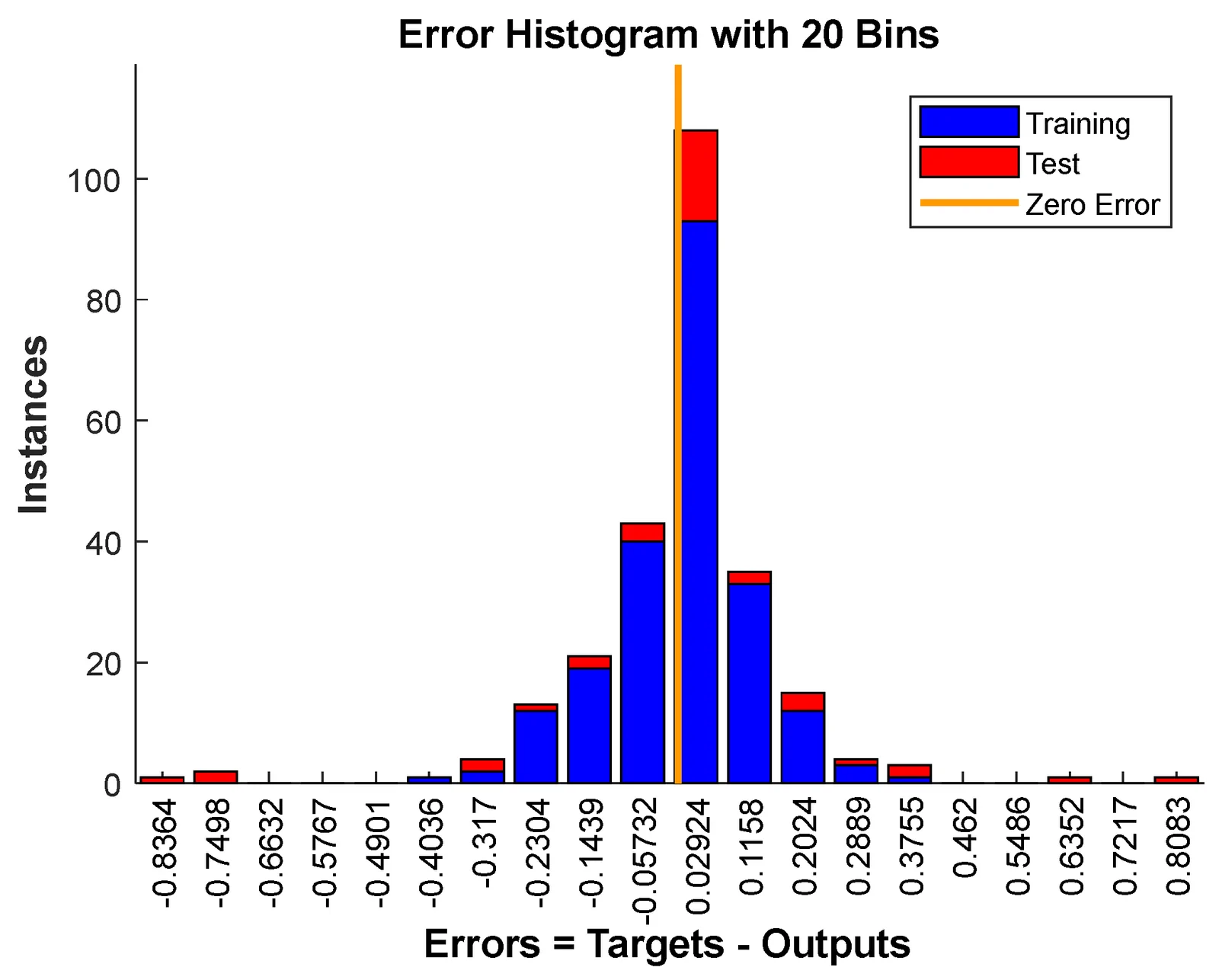
BR training algorithm error histogram.

Upon examining the error histogram for the BR algorithm shown in Figure 11, it can be seen that the majority of prediction errors are concentrated around zero. The fact that the errors are clustered within a narrow range indicates that the model predictions are quite close to the experimental results. The symmetrical distribution of the histogram demonstrates that there is no systematic bias in the model. The balance between positive and negative error values demonstrates that the developed model can maintain a similar level of accuracy across different PUBs. In particular, the very low frequency of high error values indicates that the BR algorithm can successfully model different output parameters simultaneously. This is one of the key indicators supporting the reliability of the multi-output ANN model for dataset.

**Figure 12:**
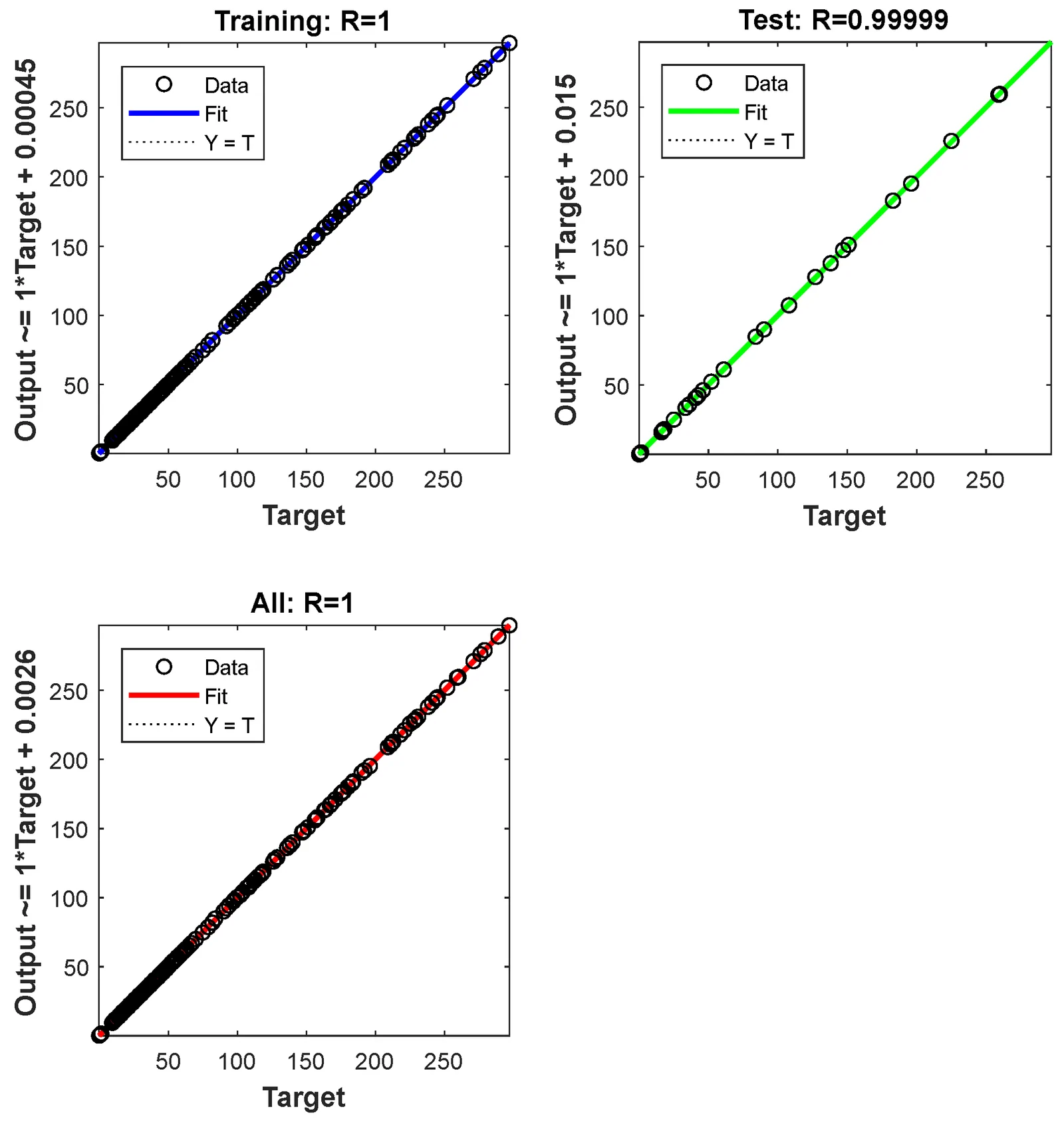
BR training algorithm error histogram. In the regression equations, the asterisk (*) symbol denotes the multiplication operator.

An examination of the regression graphs shown in Figure 12 reveals that the experimental values and the ANN predictions exhibit a nearly linear relationship. The majority of the predicted values lie on the ideal line or very close to it. This indicates that the model not only learned from the training data but is also capable of making successful predictions on independent samples for dataset. The fact that the training and test performance are quite close to each other indicates that the BR algorithm possesses strong generalization capabilities. The high correlation coefficients obtained in the regression analysis confirm that there is a very strong relationship between the model outputs and the experimental measurements. These results demonstrate that the developed network architecture can reliably predict the electrical, thermal, and mechanical properties of PUBs for selected data.

### 3.3. SCG Training Algorithm Performance Results

The developed multi-output ANN was trained using the SCG algorithm to evaluate its effectiveness in predicting the material properties of the produced PUBs. The experimental dataset consisted of 42 observations, which were randomly divided into three subsets comprising 30 samples for training, 6 samples for validation, and 6 samples for independent testing. During the training process, the SCG algorithm optimized the network weights through an iterative conjugate gradient approach that avoids the explicit computation of second-order derivatives, thereby reducing computational complexity while maintaining stable convergence. The predictive capability of the model was comprehensively assessed using the MSE, RMSE, MAE, MAPE, R, regression analysis, error histogram, learning performance curves, and independent test results. The obtained statistics demonstrated that the SCG-based ANN successfully captured the nonlinear relationships between the formulation variables and the six output responses, producing high correlation coefficients and low prediction errors for the training, validation, and testing datasets. Although its prediction accuracy was slightly lower than that achieved by the BR and LM algorithms, the SCG algorithm nevertheless exhibited reliable convergence behavior, satisfactory generalization capability, and consistent predictive performance, confirming its suitability as an efficient alternative for modeling the electrical, thermal, physical, and mechanical properties of the developed PUBs [55].

**Figure 13:**
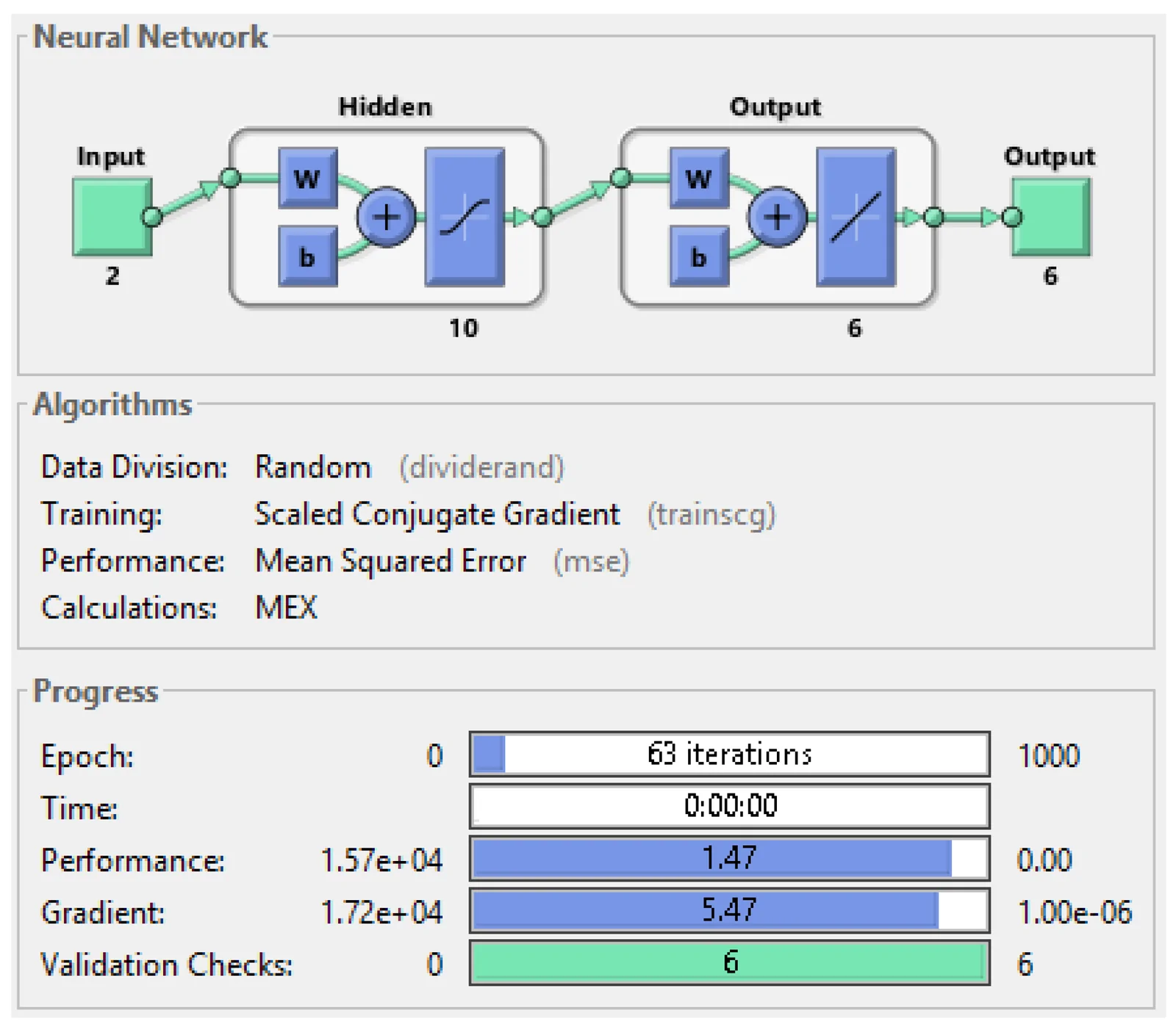
SCG training algorithm: ANN architecture and results.

The SCG algorithm is an improved version of the classic Conjugate Gradient method and is an iterative optimization approach that operates without the need to compute second derivatives. It is particularly preferred for large datasets due to its low computational cost and limited memory usage. However, it is known that prediction accuracy in small and medium-sized datasets can vary depending on the problem at hand. Figure 13 presents the network architecture and performance results obtained using the SCG algorithm for dataset.

**Figure 14:**
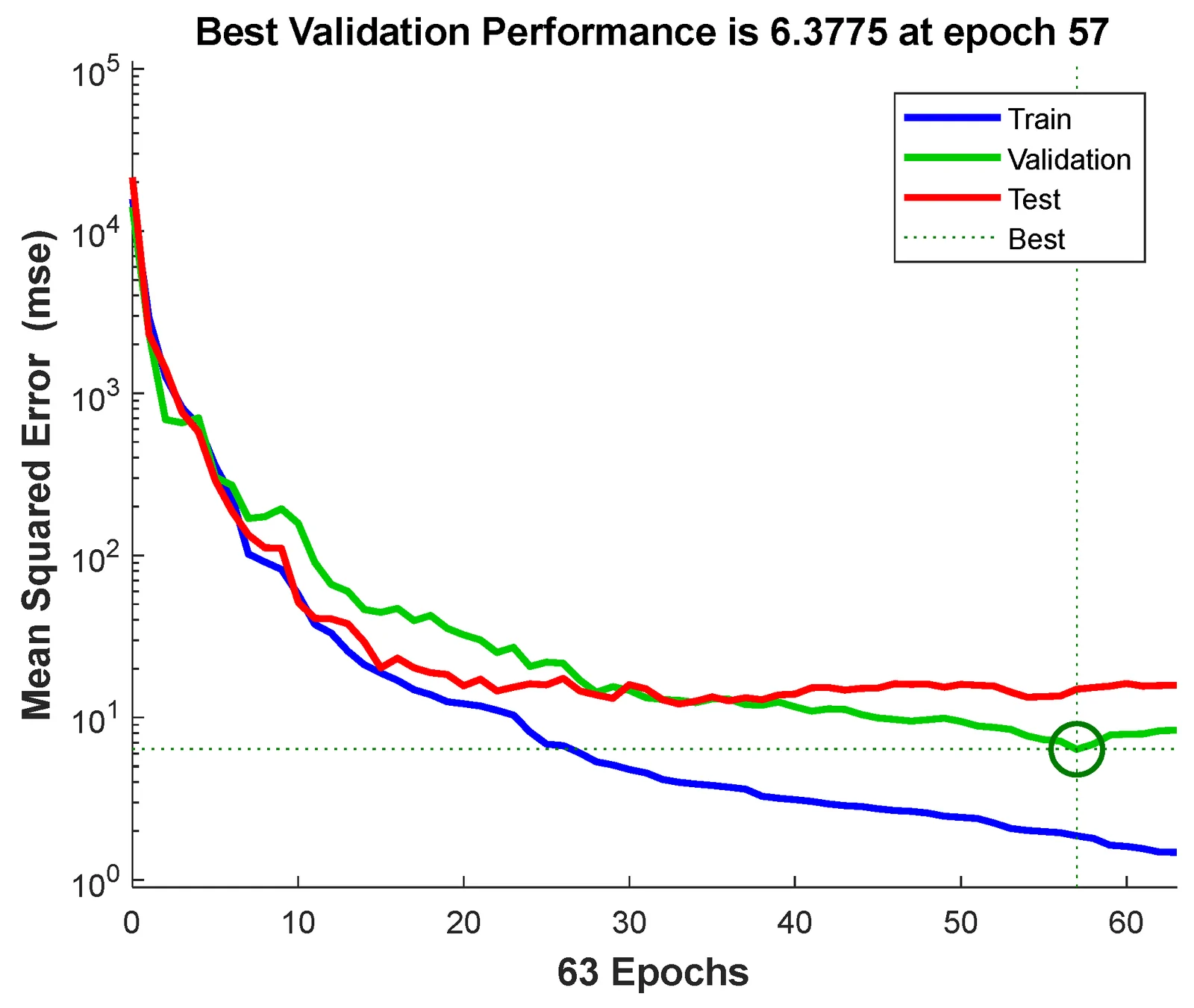
SCG training algorithm performance curves.

Figure 14 shows that the error function generally tends to decrease during the training process carried out using the SCG algorithm. However, it is noteworthy that the rate of error reduction is slower compared to the LM and BR algorithms. Although the optimization process progressed steadily during training, the SCG algorithm was less successful than the other two algorithms in reaching the minimum error value for dataset.

**Figure 15:**
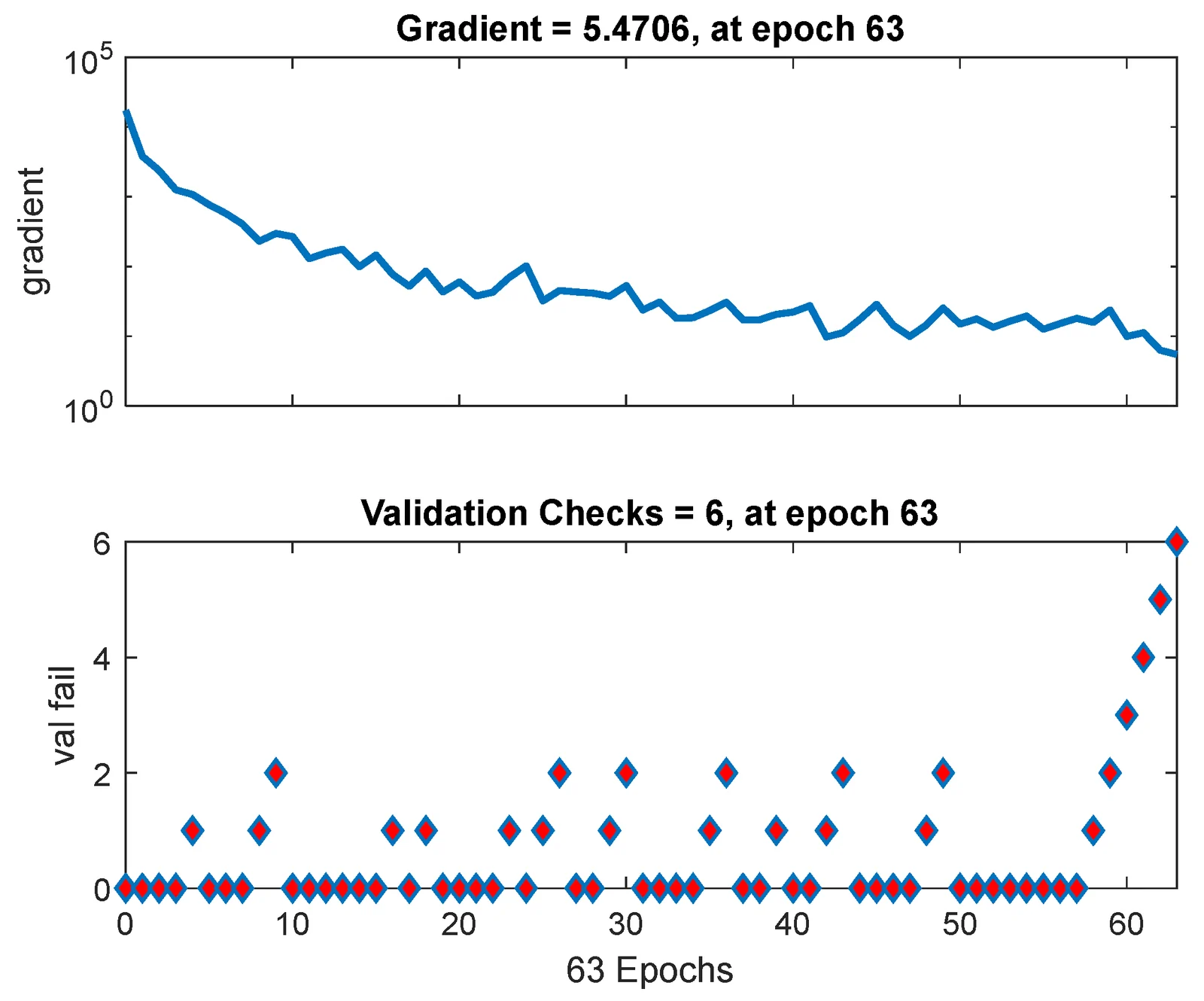
SCG training algorithm: gradient and validation check plots.

As can be seen from the data in Figure 15, although there was a decrease in the gradient value at epoch 63, it is evident that a satisfactory level was not achieved compared to the other two learning methods. Furthermore, it is clear that a sufficiently good result was not obtained in the validation test.

**Figure 16:**
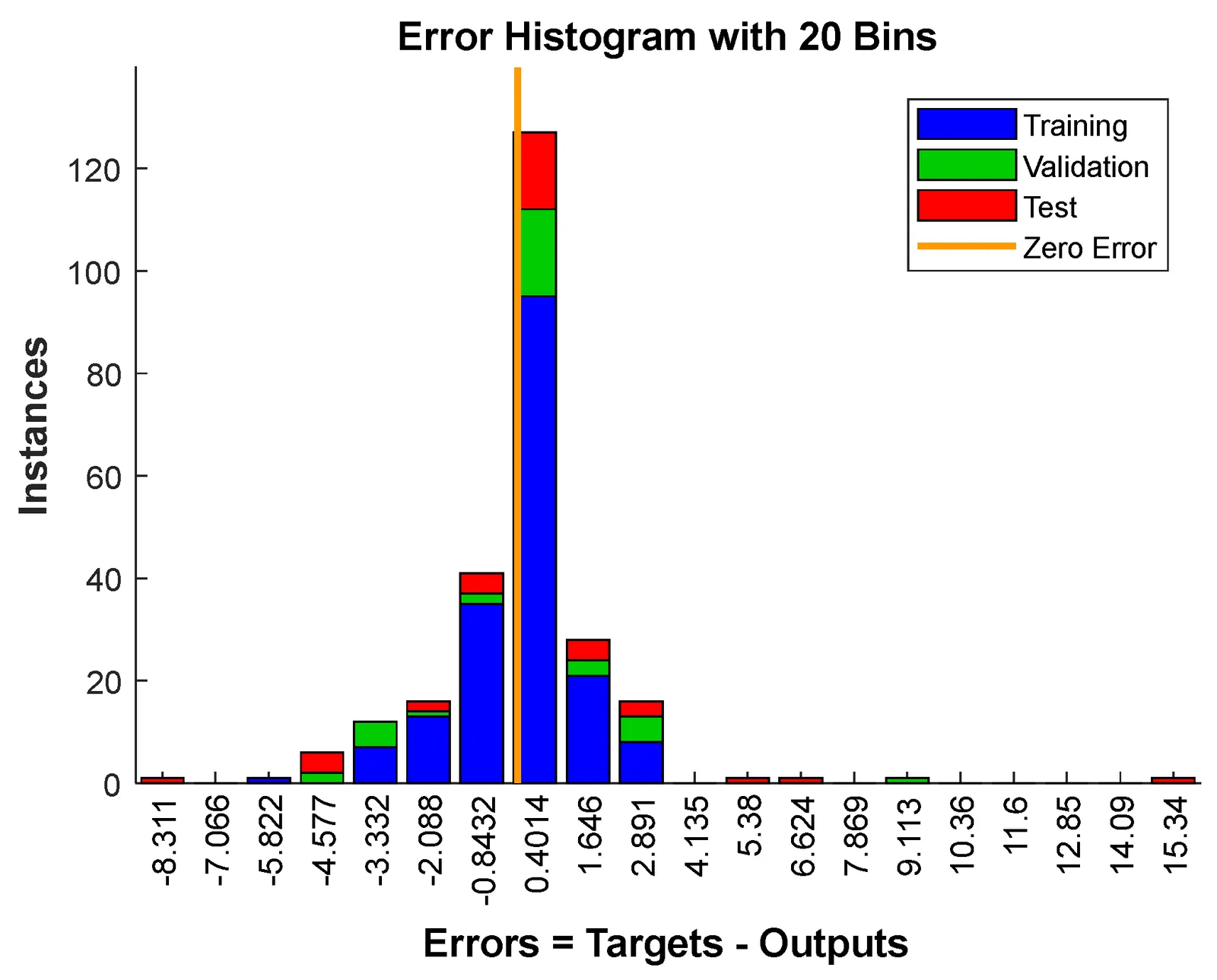
SCG training algorithm error histogram.

Upon examining the error histogram for the SCG algorithm shown in Figure 16, it can be seen that the majority of the error values are again concentrated around zero. However, the spread of the histogram is wider than that of the LM and, in particular, the BR algorithms. This indicates an increase in the variance of the prediction errors. In other words, while predictions for some PUBs will be quite close to the experimental results, the error will reach higher levels in other samples for dataset. Although the overall structure of the histogram shows that the model does not introduce systematic bias, the wide error distribution indicates that prediction accuracy will be lower compared to the other two algorithms for selected dataset.

**Figure 17:**
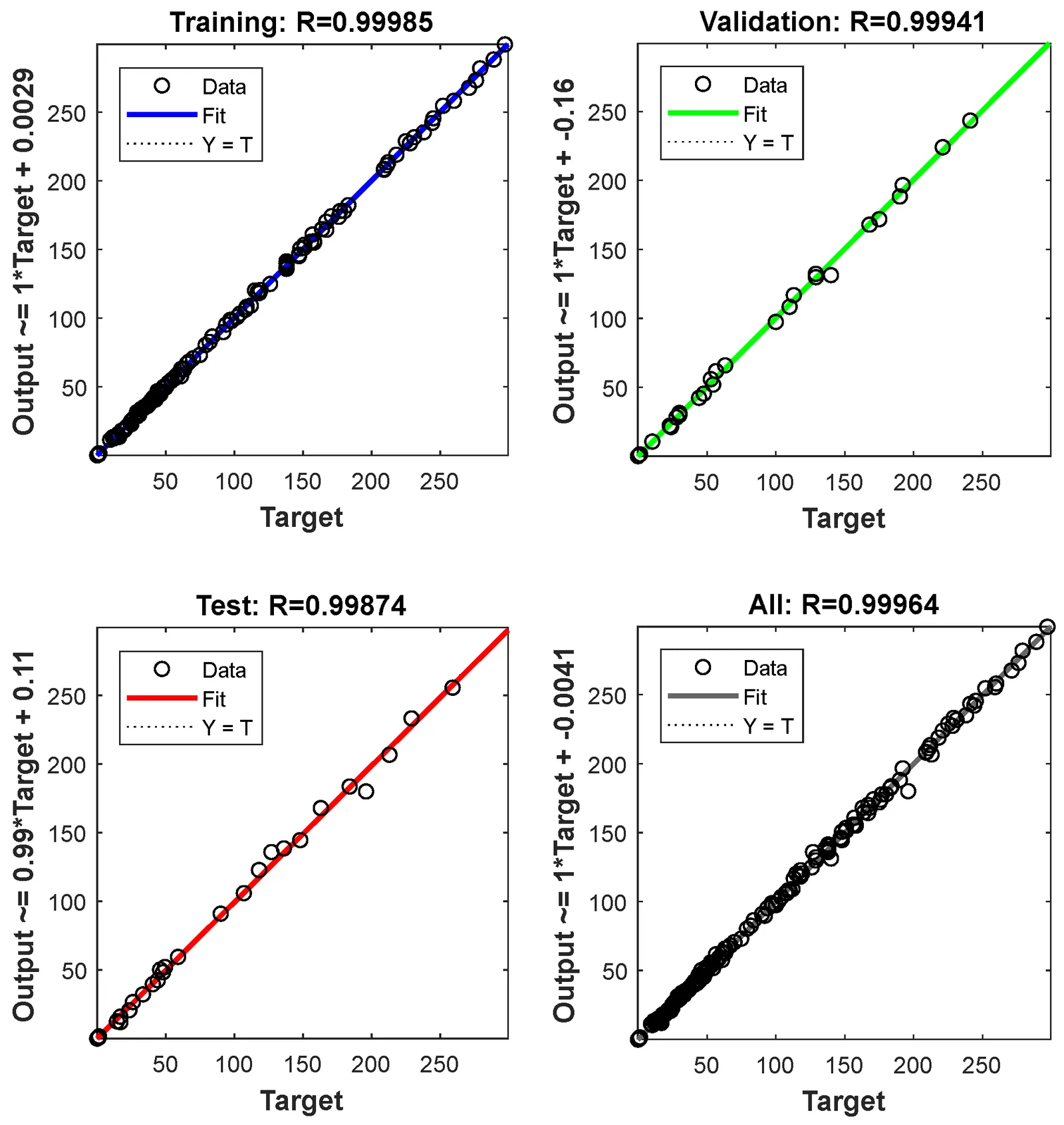
SCG training algorithm regression results. In the regression equations, the asterisk (*) symbol denotes the multiplication operator.

The regression plots show a strong linear relationship between the experimental values and the artificial neural network predictions. While a significant portion of the predicted points lie on the ideal regression line, Figure 17 shows that the scatter is greater compared to the LM and BR algorithms. This indicates that prediction accuracy may decrease in some cases. The absence of significant performance differences between the training and test datasets indicates that the model possesses a certain level of generalization ability. However, the fact that the deviation of the regression lines from the ideal line is slightly greater than that of the other two algorithms clearly highlights the difference in prediction performance for dataset.

One of the most important indicators of a model’s success is its performance on independent test data that was not used during training. Since the BR approach is expected to yield better results on small datasets, it is believed that it will produce consistent predictions on independent data for selected data.

### 3.4. Algorithm Performance Analysis Results

**Figure 18:**
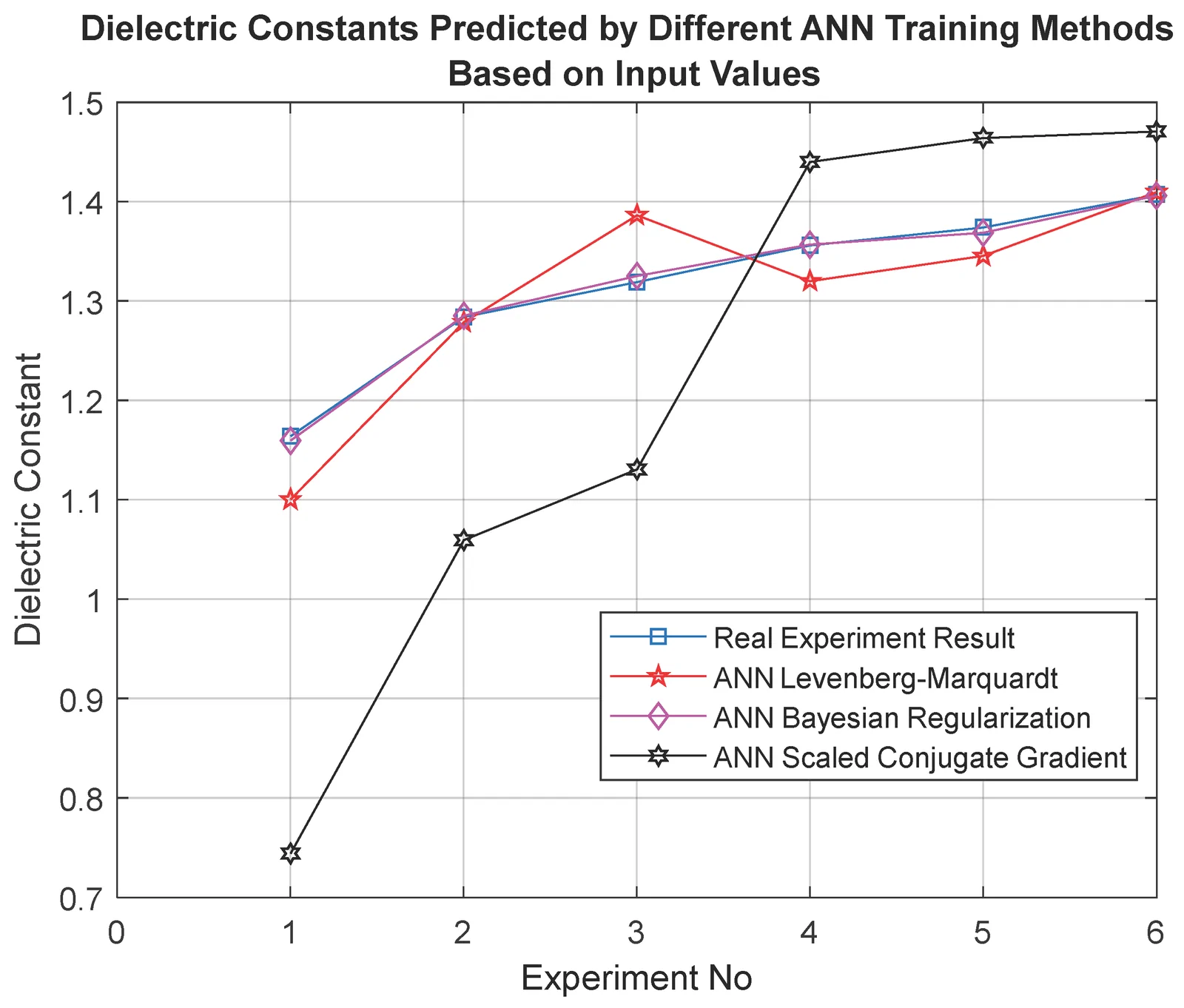
Dielectric constants predicted using different ANN training methods.

The inputs from six independent datasets (WAS % and MSO %) were applied to ANN architectures trained using three different training algorithms. As a result, the predicted output values generated by each network architecture were obtained. The obtained output values were plotted on graphs to facilitate a better understanding by comparing them with the actual experimental results in dataset. Figure 18 shows graphs of the prediction results from the training algorithms in each network architecture and the actual experimental results for the first output value, the dielectric constant. Upon examining the graph, it can be clearly stated that the BR method achieved the best prediction value for six independent data in datasets.

**Figure 19:**
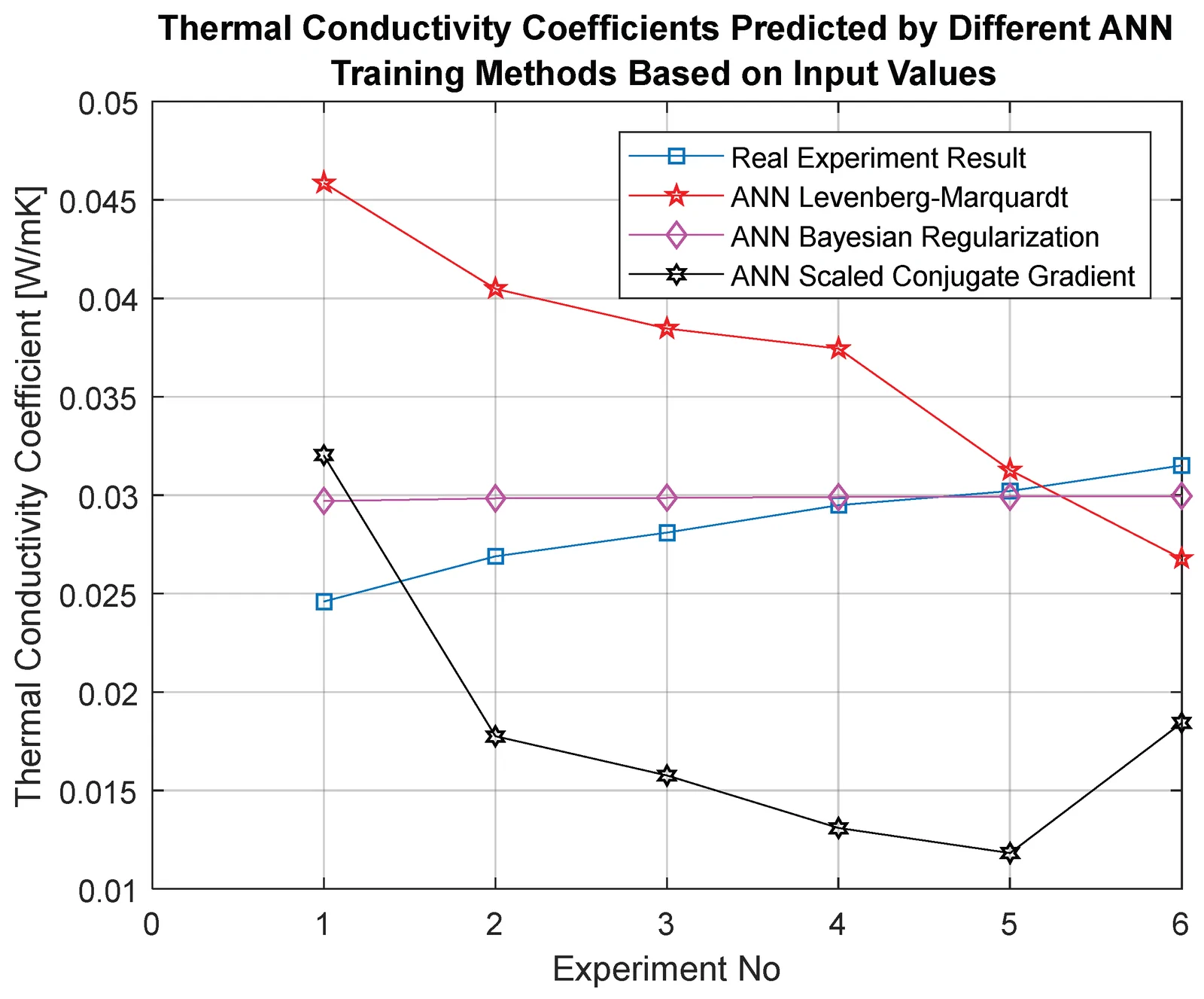
Thermal conductivity coefficients predicted using different ANN training methods.

The second output values are the thermal conductivity coefficients (W/m·K). Figure 19 shows graphs of the output values obtained when independent data inputs were applied to the network structures resulting from training sessions conducted on the same network architecture using different training algorithms. Upon examining the actual output values for the six independent variables, it can be concluded—as seen in the graphs—that the BR method produced the closest estimate for six independent data in datasets.

**Figure 20:**
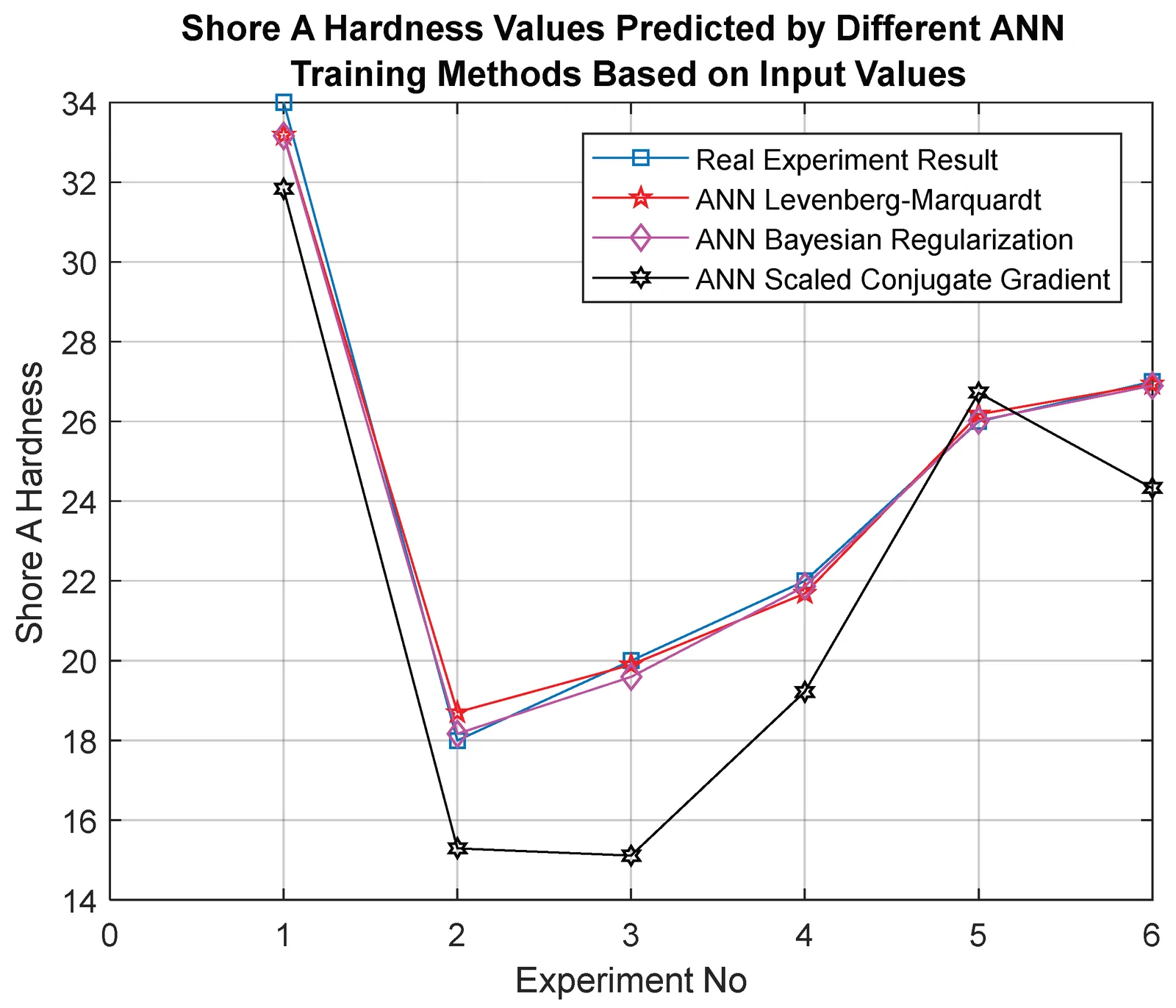
Shore A hardness values predicted using different ANN training methods.

The Shore A hardness value constitutes another output value in the dataset. The input values of six independent variables were applied separately to the LM network architecture, the BR network architecture, and the SCG network architecture. The resulting Shore A hardness output values were plotted after being compared with actual experimental results. An examination of the graphs in Figure 20 reveals that both the LM and BR methods produced successful results, moving in close proximity to one another and providing predictions close to the actual output values in dataset.

**Figure 21:**
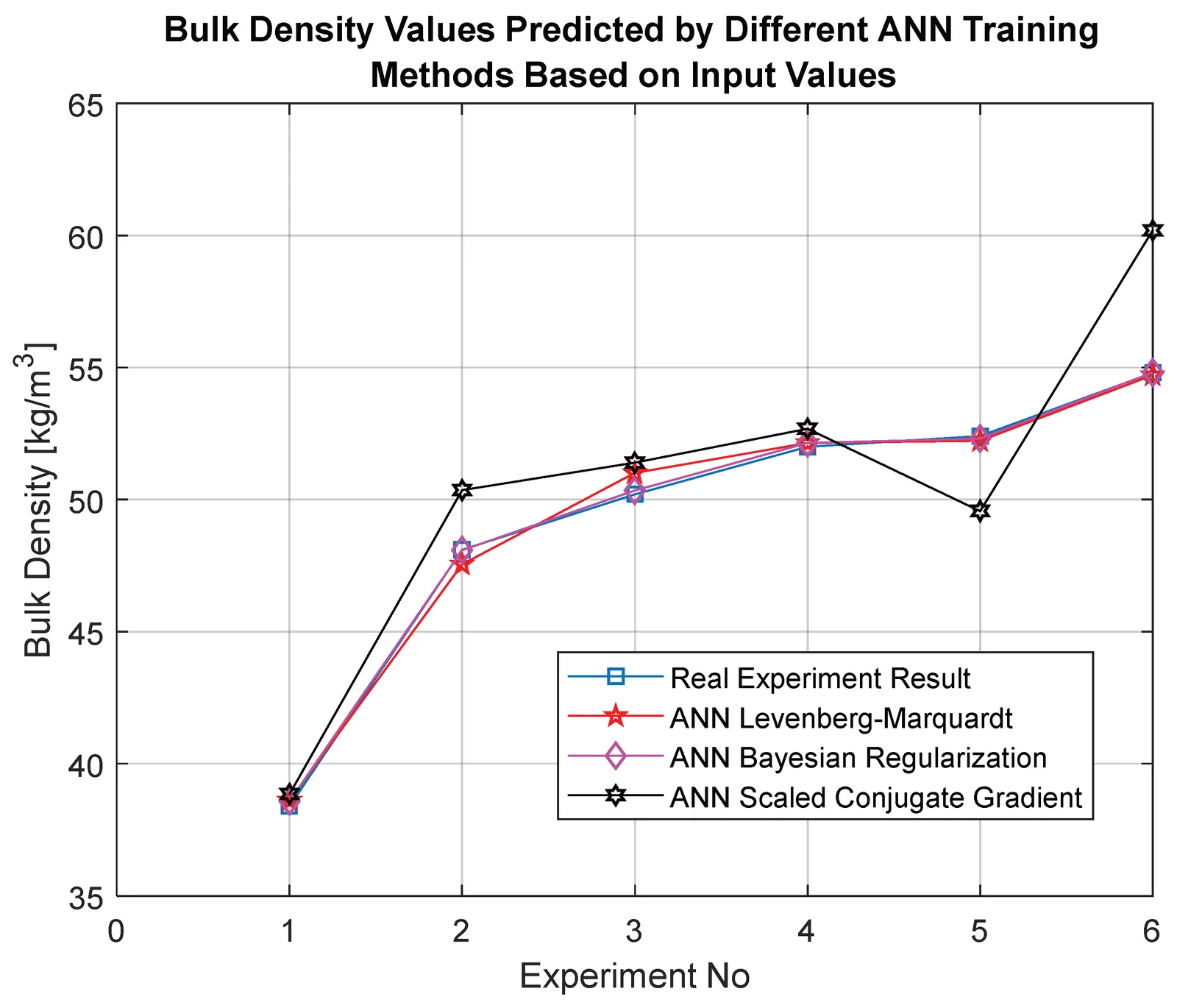
Bulk density values predicted using different ANN training methods.

Figure 21 shows graphs of the predicted bulk density output values obtained by applying the input values of six independent data points to ANN trained using three different training algorithms for dataset. When compared with the actual bulk density output values, it can be said that the best prediction result was achieved using the BR method.

**Figure 22:**
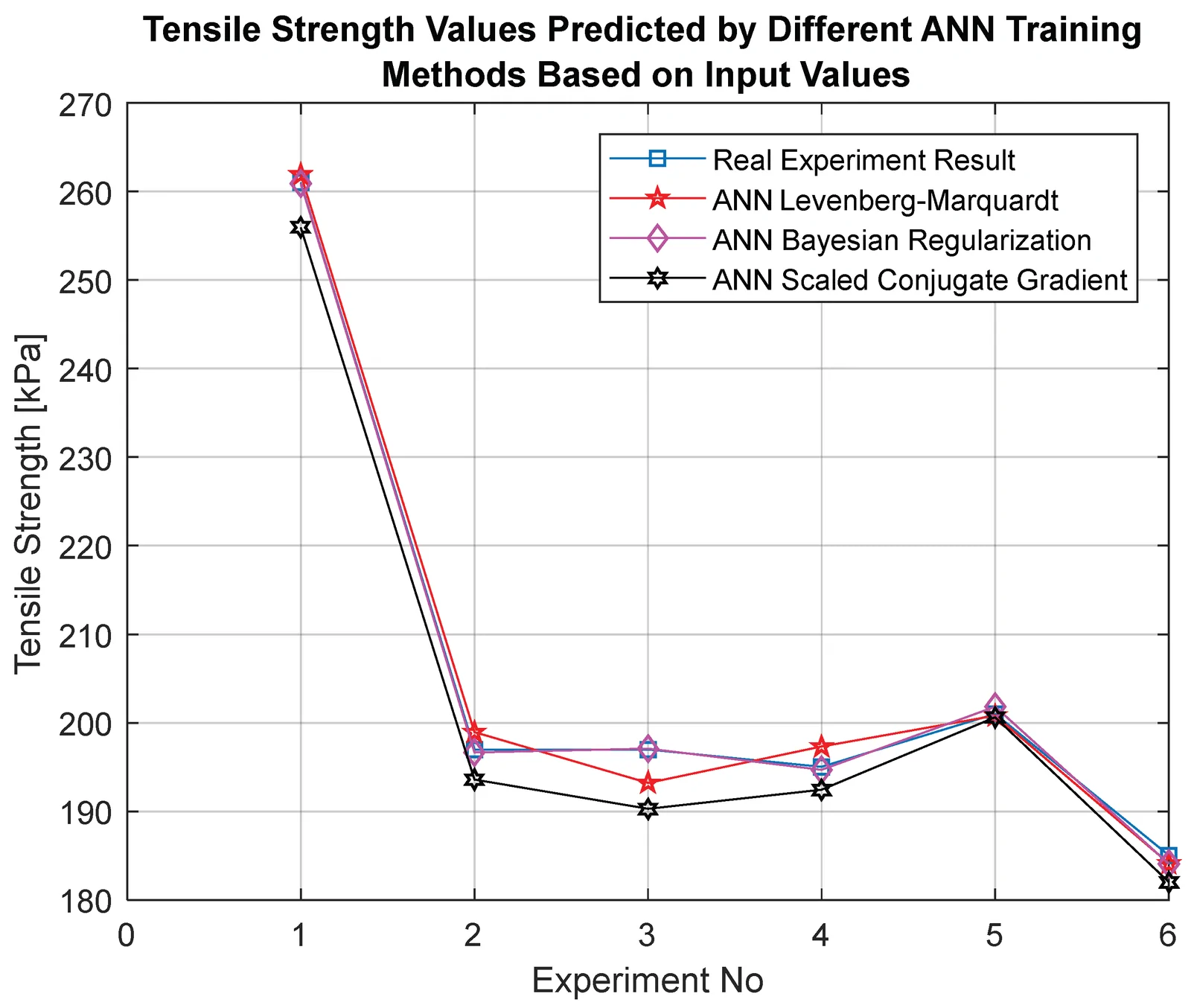
Tensile strength values predicted using different ANN training methods.

Figure 22 shows the ANN prediction graphs for tensile strength, another output value in dataset. The input values from 6 independent data sets were applied to networks trained by changing only the training algorithms, without altering the network architecture. The tensile strength values predicted using each network architecture were plotted on the graph to allow comparison with the actual experimental output values in dataset.

**Figure 23:**
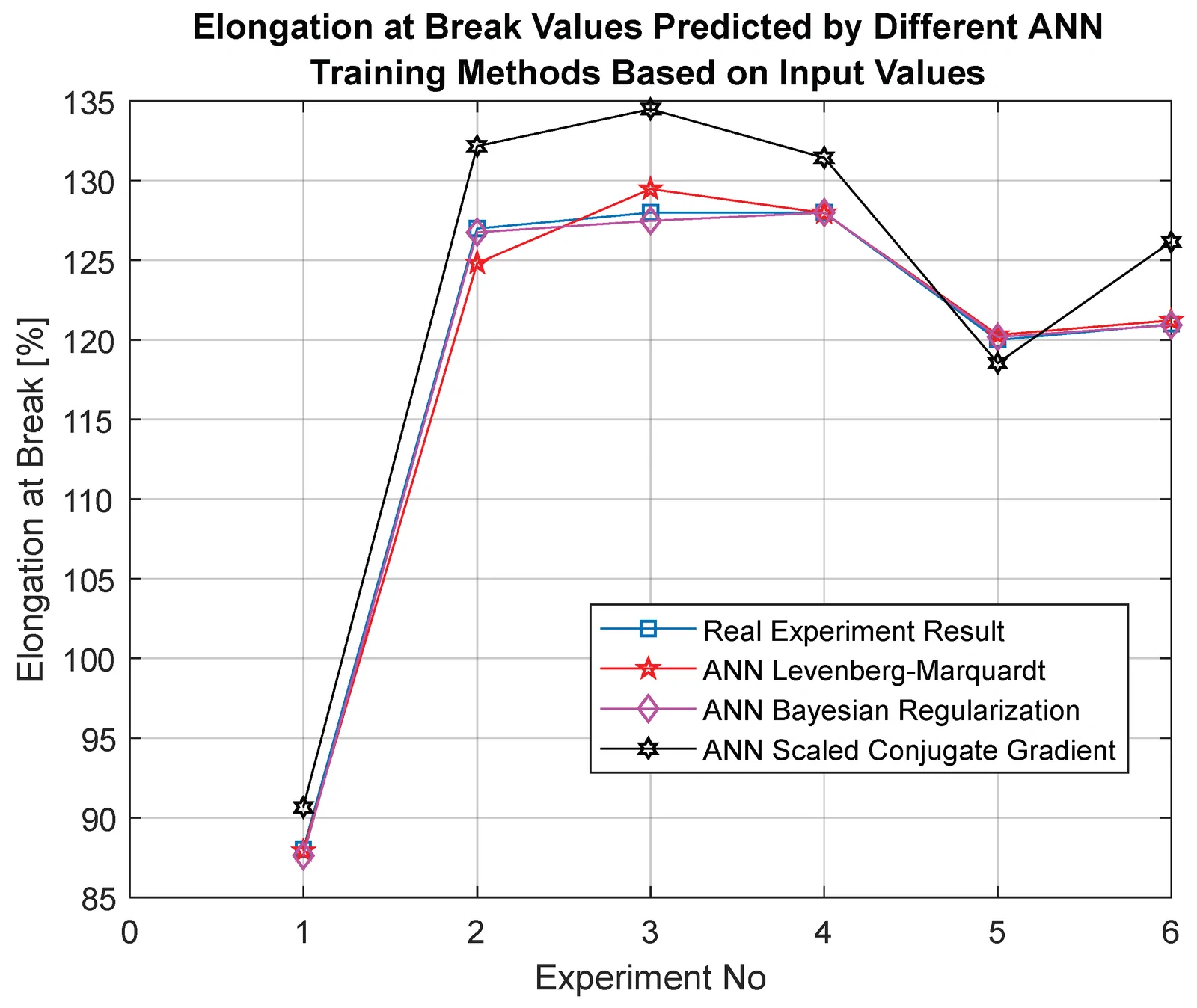
Elongation at break values predicted using different ANN training methods.

The final output of the network architecture is the elongation at break value in the dataset. Input values from independent data sets were applied to ANNs of the same architecture trained using different training algorithms, and estimated elongation at break values were obtained. To enable comparison with the actual values, the results produced by the ANNs trained using different algorithms were visualized and plotted as shown in Figure 23. Upon examining the graphs, it is evident that the best prediction result was achieved using the BR method for dataset.

In general, a rough assessment of which method was more successful was made by examining the graphs of the output values in dataset. To clearly demonstrate success, the estimated output values from the independent data for each method were entered into tables. The tables provide the estimated and actual values for six output values, along with the error values, MSE, RMSE, MAE and MAPE. Table 2 presents the numerical values of the estimated and actual output values for three different methods for dataset.

Table 3 presents the error values resulting from the estimated values obtained using each method. Based on these error values, MSE, RMSE, MAE and MAPE for each method were determined.

Based on the errors in Table 3, MSE, RMSE, MAE and MAPE—which will be considered performance criteria—were calculated. When examining the performance of the three different ANN training algorithms, it is observed that the worst result was obtained with the SCG method. Although the LM method did not produce particularly poor results in its predictions, the BR method demonstrated the best prediction performance. Among the three ANN training algorithms, the BR method proved to be the most suitable for this dataset. The BR method was able to predict six different output values with higher accuracy.

The development of PUBs requires lengthy experimental processes. Preparing samples, performing the operations, and conducting mechanical tests for each new filler ratio impose a significant burden in terms of both time and cost. Thanks to the ANN model developed in this study, six different material properties could be predicted simultaneously with high accuracy using only two input parameters for dataset. This demonstrates that experimental work can be significantly reduced. In particular, the BR algorithm’s ability to produce reliable predictions for different filler ratios will make significant contributions to AI-supported optimization studies in biocomposite design in the future. This comparison demonstrates that, in terms of the dataset used and the developed multi-output artificial neural network model, the BR algorithm is the most successful training method for this dataset, the LM algorithm ranks second, and the SCG algorithm lags behind the other two methods [56].

### 3.5. Experimental Results

**Figure 24:**
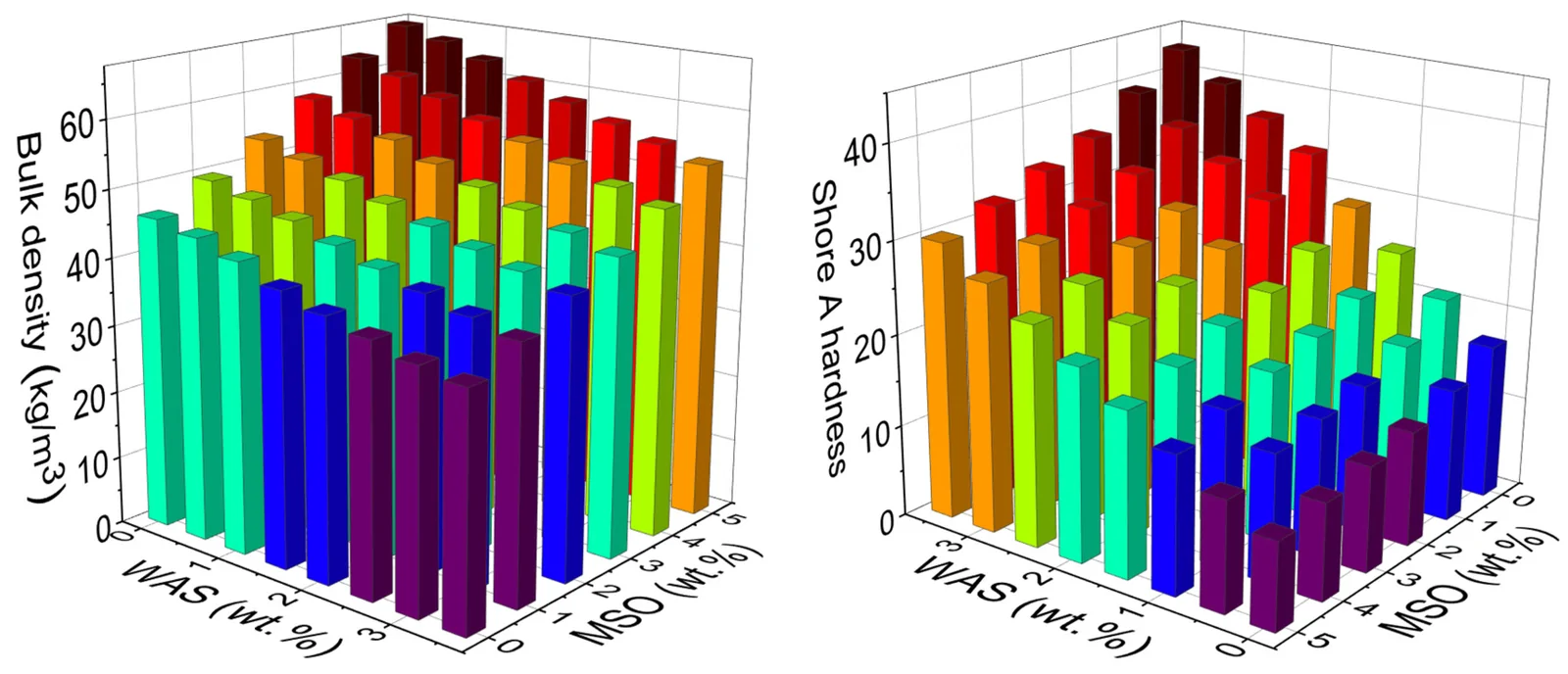

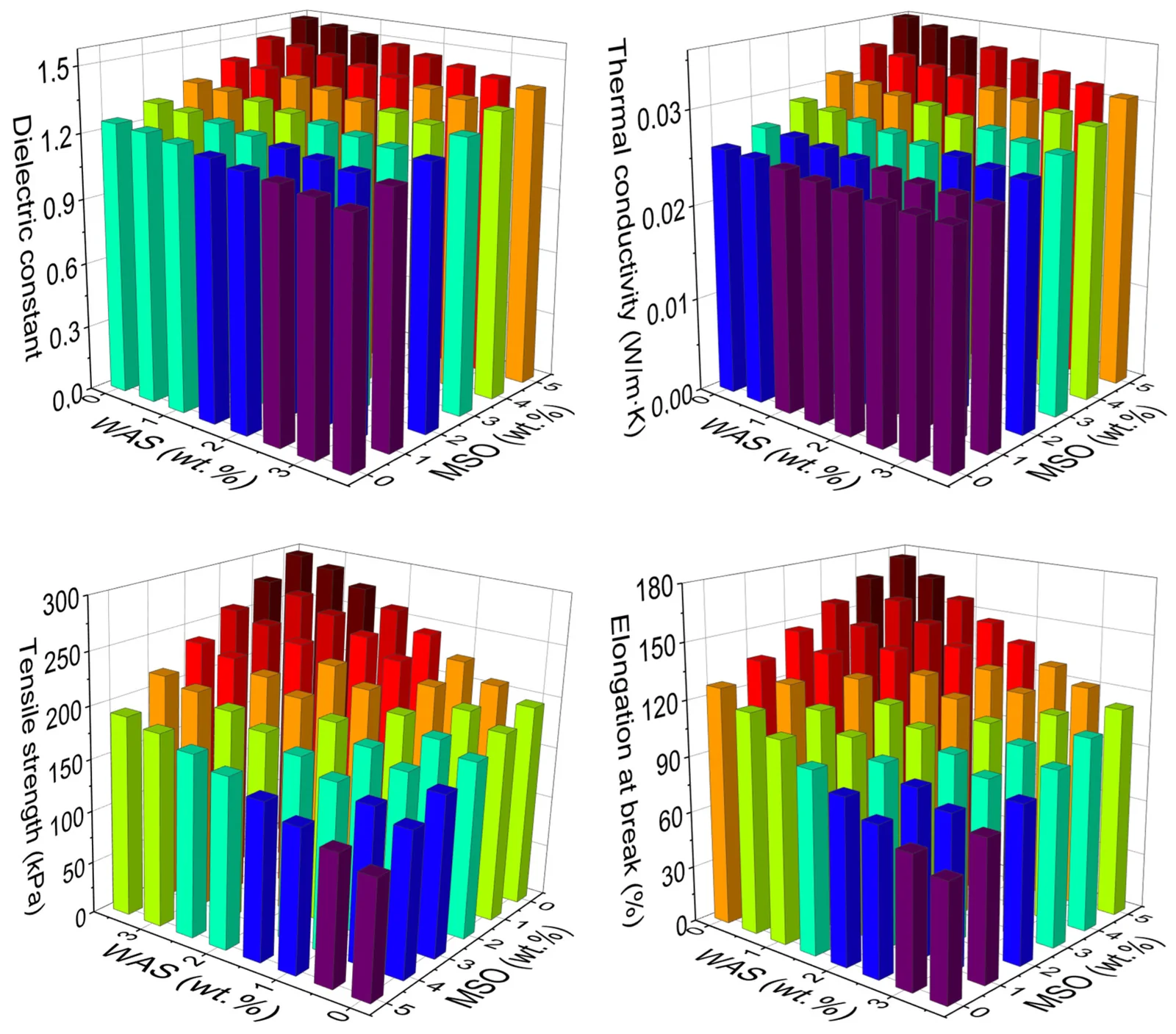
Experimental results of the physical, mechanical, thermal, and electrical properties of PUBs as a function of WAS and MSO content.

The results demonstrate that variations in WAS and MSO contents were associated with pronounced and combined changes in the electrical, thermal, physical, and mechanical properties of the produced PUBs (Figure 24). As illustrated by the three-dimensional response plots, the measured properties exhibited distinct nonlinear trends across the investigated composition range, suggesting that the combined incorporation of lignocellulosic reinforcement and bio-based modification influenced the overall behavior of the PUBs in a non-linear manner. Bulk density gradually increased with increasing MSO content, while moderate additions of WAS were also accompanied by a slight increase in density, consistent with the incorporation of lignocellulosic particles into the polyurethane matrix. This trend may be related to changes in the internal structure and distribution of the incorporated phases within the composite. Shore A hardness generally increased with increasing MSO concentration and moderate WAS incorporation, indicating a more pronounced hardness response within this formulation range. However, further increases in WAS content were accompanied by a slight decrease in hardness. This variation may be associated with changes in the dispersion and distribution of WAS within the polymer matrix, which could influence the local structure and mechanical response of the composite. The observed variations in density and hardness reflect the combined influence of WAS and MSO contents on the physical and mechanical characteristics of the developed PUBs [57].

The dielectric constant increased progressively with increasing MSO content and exhibited a moderate dependence on WAS loading. This behavior is associated with enhanced interfacial polarization resulting from the introduction of polar functional groups present in both the MSO and the lignocellulosic constituents of WAS. The greater number of heterogeneous interfaces promoted dipole orientation under the applied electric field, leading to improved dielectric polarization of the composite system. A similar increasing tendency was observed for thermal conductivity, where both constituents contributed to more efficient heat transfer through the composite structure. The gradual increase in thermal conductivity indicates that the incorporation of WAS and MSO improved thermal transport pathways within the cross-linked polyurethane network without causing abrupt structural discontinuities.

The mechanical properties showed a non-monotonic response to changes in MSO content. Tensile strength increased with increasing MSO concentration up to intermediate formulation levels, followed by a decrease at higher MSO contents. This variation may be related to changes in the dispersion and distribution of the incorporated phase within the polymer matrix, which could affect the overall mechanical response of the developed PUBs. Nevertheless, at the highest WAS and MSO contents, tensile strength exhibited a slight decline, which is likely associated with filler agglomeration, reduced matrix continuity, and the formation of localized stress concentration sites that facilitate crack initiation during loading. In contrast, elongation at break generally increased with increasing MSO content because the bio-based modifier enhanced polymer chain mobility and reduced the intrinsic brittleness of the polyurethane network. Moderate amounts of WAS maintained adequate ductility while simultaneously reinforcing the matrix, whereas excessive reinforcement restricted molecular mobility and slightly reduced deformation capability [58].

The experimental findings demonstrate that the simultaneous incorporation of WAS and MSO enables effective tailoring of multiple functional properties of the produced PUBs. The figures indicate favorable formulation regions in which electrical insulation characteristics, thermal transport behavior, density, hardness, tensile strength, and ductility exhibit a balanced combination of properties. These findings highlight the potential of appropriately adjusting the WAS and MSO contents to achieve a desirable balance among the various functional and mechanical properties of the developed PUBs. These complex nonlinear interactions further justify the application of multi-output ANN modeling; as conventional regression techniques are unlikely to capture the coupled effects of both formulation variables with comparable accuracy. The experimental dataset provides a robust foundation for developing predictive artificial intelligence models capable of accelerating material design and optimization while substantially reducing experimental time, material consumption, and development costs [59].

## 4. Conclusions

In this study, sustainable PUBs incorporating WAS and MSO are successfully developed, and their electrical, thermal, physical, and mechanical properties are systematically investigated. A total of 48 formulations are prepared by varying the WAS content from 0 to 3.5 wt.% and the MSO content from 0 to 5 wt.%. The experimental results demonstrate that changes in both formulation variables are associated with substantial variations in the investigated properties. The dielectric constant varies from 1.10 to 1.56, while the thermal conductivity ranges from 0.024 to 0.036 W m^−1^ K^−1^. Shore A hardness varies between 9.5 and 43.0, and bulk density ranges from 34 to 67 kg m^−3^. Tensile strength and elongation at break vary from 115 to 297 kPa and from 62 to 176%, respectively. These results demonstrate that the electrical, thermal, physical, and mechanical characteristics of the PUBs can be tailored through appropriate adjustment of WAS and MSO contents.

The experimental trends further indicate that increasing WAS content generally decreases the dielectric constant, thermal conductivity, bulk density, and elongation at break, while increasing Shore A hardness and tensile strength. In contrast, increasing MSO content generally increases the dielectric constant, thermal conductivity, bulk density, and elongation at break, whereas it decreases Shore A hardness and tensile strength. The highest dielectric constant (1.560), bulk density (67.0 kg m^−3^), elongation at break (176%), and thermal conductivity (0.0360 W m^−1^ K^−1^) are obtained at the formulation containing 0 wt.% WAS and 5 wt.% MSO. In comparison, the highest Shore A hardness (43.0) and tensile strength (297 kPa) are obtained at 3.5 wt.% WAS and 0 wt.% MSO, whereas the lowest bulk density (34 kg m^−3^) and thermal conductivity (0.0240 W m^−1^ K^−1^) occur at the same WAS-rich and MSO-free formulation. These findings demonstrate the presence of clear formulation-dependent trade-offs among stiffness, strength, flexibility, density, thermal transport, and dielectric behavior.

The ANN analysis provides an effective computational framework for describing these formulation–property relationships using WAS and MSO contents as input variables and the six experimentally measured properties as simultaneous outputs for dataset. Among the investigated training algorithms, BR is identified as the most promising model, providing stronger predictive and generalization capability than the LM and SCG algorithms for dataset. The close agreement between the experimental results and the predictions obtained from the best-performing ANN model demonstrates the applicability of ANN-based modeling for simultaneous prediction of the multifunctional properties of the developed PUBs. Importantly, the model enables the complex effects of two formulation variables on six different material responses to be evaluated without requiring a separate predictive model for each property.

Therefore, the combination of WAS, MSO, PUBs fabrication, and ANN-assisted property prediction provides an efficient strategy for the development of sustainable multifunctional polymeric materials. The developed experimental–computational approach can reduce the need for extensive formulation trials and facilitate rapid screening and future optimization of PUB compositions for applications requiring tailored electrical, thermal, physical, and mechanical performance. Further studies incorporating additional structural and interfacial characterization could provide deeper insight into the molecular and microstructural mechanisms governing the observed formulation–property relationships.

## Figures and Tables

**Table 1:** Formulations of PUBs prepared with different WAS and MSO contents.

Exp.	WAS (wt.%)	MSO (wt.%)	Exp.	WAS (wt.%)	MSO (wt.%)	Exp.	WAS (wt.%)	MSO (wt.%)
1	0.0	0	17	0.0	2	33	0.0	4
2	0.5	0	18	0.5	2	34	0.5	4
3	1.0	0	19	1.0	2	35	1.0	4
4	1.5	0	20	1.5	2	36	1.5	4
5	2.0	0	21	2.0	2	37	2.0	4
6	2.5	0	22	2.5	2	38	2.5	4
7	3.0	0	23	3.0	2	39	3.0	4
8	3.5	0	24	3.5	2	40	3.5	4
9	0.0	1	25	0.0	3	41	0.0	5
10	0.5	1	26	0.5	3	42	0.5	5
11	1.0	1	27	1.0	3	43	1.0	5
12	1.5	1	28	1.5	3	44	1.5	5
13	2.0	1	29	2.0	3	45	2.0	5
14	2.5	1	30	2.5	3	46	2.5	5
15	3.0	1	31	3.0	3	47	3.0	5
16	3.5	1	32	3.5	3	48	3.5	5

**Table 2:** Actual output values and estimated output values.

**No.**	**Real Outputs**					
	Dielectric Constant	Thermal Conductivity (W/m·K)	Shore A Hardness	Bulk Density (kg/m^3^)	Tensile Strength (kPa)	Elongation at Break (%)
1	1.164	0.0246	34	38.4	261	88
2	1.284	0.0269	18	48.1	197	127
3	1.319	0.0281	20	50.2	197	128
4	1.356	0.0295	22	52	195	128
5	1.374	0.0302	26	52.4	201	120
6	1.407	0.0315	27	54.8	185	121
	Outputs Predicted using ANN Levenberg–Marquardt					
	Dielectric Constant	Thermal Conductivity (W/m·K)	Shore A Hardness	Bulk Density (kg/m^3^)	Tensile Strength (kPa)	Elongation at Break (%)
1	1.1002	0.0459	33.1798	38.6168	261.9014	87.8948
2	1.2787	0.0405	18.7026	47.5524	198.9283	124.8210
3	1.3863	0.0385	19.8916	51.0028	193.1969	129.4672
4	1.3200	0.0374	21.6824	52.1430	197.2983	127.9631
5	1.3452	0.0313	26.1778	52.2218	200.7872	120.3139
6	1.4094	0.0268	26.9373	54.7111	184.0879	121.2235
	Outputs Predicted using ANN Bayesian Regularization					
	Dielectric Constant	Thermal Conductivity (W/m·K)	Shore A Hardness	Bulk Density (kg/m^3^)	Tensile Strength (kPa)	Elongation at Break (%)
1	1.1594	0.0297	33.1572	38.5798	260.9034	87.6028
2	1.2852	0.0298	18.1624	48.0848	196.6441	126.7617
3	1.3250	0.0299	19.5972	50.3383	197.0629	127.4929
4	1.3565	0.0299	21.8666	52.1470	194.6655	128.0075
5	1.3686	0.0299	26.0213	52.3113	201.8220	120.1685
6	1.4058	0.0300	26.8960	54.8112	184.0857	120.9365
	Outputs Predicted using ANN Scaled Conjugate Gradient					
	Dielectric Constant	Thermal Conductivity (W/m·K)	Shore A Hardness	Bulk Density (kg/m^3^)	Tensile Strength (kPa)	Elongation at Break (%)
1	0.7440	0.0320	31.8288	38.8490	255.9505	90.6497
2	1.0594	0.0178	15.2943	50.3572	193.5838	132.1695
3	1.1307	0.0158	15.1097	51.3929	190.2663	134.4981
4	1.4395	0.0131	19.2188	52.6798	192.4136	131.4488
5	1.4641	0.0118	26.7194	49.5699	200.6117	118.5422
6	1.4704	0.0184	24.3261	60.1931	181.9844	126.1579

**Table 3:** Estimated output value errors and performance calculations for different ANN training algorithms.

**No.**	ANN Levenberg–Marquardt Errors					
	Dielectric Constant	Thermal Conductivity (W/m·K)	Shore A Hardness	Bulk Density (kg/m^3^)	Tensile Strength (kPa)	Elongation at Break (%)
1	0.0638	0.0213	0.8202	0.2168	0.9014	0.1052
2	0.0053	0.0136	0.7026	0.5476	1.9283	2.1790
3	0.0673	0.0104	0.1084	0.8028	3.8031	1.4672
4	0.0360	0.0079	0.3176	0.1430	2.2983	0.0369
5	0.0288	0.0011	0.1778	0.1782	0.2128	0.3139
6	0.0024	0.0047	0.0627	0.0889	0.9121	0.2235
MSE	0.0018	0.0001	0.2191	0.1752	4.1923	1.1769
RMSE	0.0423	0.0118	0.4681	0.4186	2.0475	1.0849
MAE	0.0339	0.0098	0.3649	0.3296	1.6760	0.7210
MAPE %	2.6530	36.5827	1.5362	0.6799	0.8387	0.5761
	ANN Bayesian Regularization Errors					
	Dielectric Constant	Thermal Conductivity (W/m·K)	Shore A Hardness	Bulk Density (kg/m^3^)	Tensile Strength (kPa)	Elongation at Break (%)
1	0.0046	0.0051	0.8428	0.1798	0.0966	0.3972
2	0.0012	0.0029	0.1624	0.0152	0.3559	0.2383
3	0.0060	0.0018	0.4026	0.1383	0.0629	0.5071
4	0.0005	0.0004	0.1334	0.1470	0.3345	0.0075
5	0.0054	0.0003	0.0213	0.0887	0.8220	0.1685
6	0.0012	0.0015	0.1040	0.0112	0.9143	0.0635
MSE	0.000015	0.000007	0.1547	0.0135	0.2939	0.0840
RMSE	0.003873	0.002646	0.3933	0.1164	0.5421	0.2899
MAE	0.00315	0.002	0.2779	0.0967	0.4310	0.2304
MAPE %	0.2431	7.5049	1.0781	0.2080	0.2207	0.2057
	ANN Scaled Conjugate Gradient Errors					
	Dielectric Constant	Thermal Conductivity (W/m·K)	Shore A Hardness	Bulk Density (kg/m^3^)	Tensile Strength (kPa)	Elongation at Break (%)
1	0.4200	0.0074	2.1712	0.4490	5.0495	2.6497
2	0.2246	0.0091	2.7057	2.2572	3.4162	5.1695
3	0.1883	0.0123	4.8903	1.1929	6.7337	6.4981
4	0.0835	0.0164	2.7812	0.6798	2.5864	3.4488
5	0.0901	0.0184	0.7194	2.8301	0.3883	1.4578
6	0.0634	0.0131	2.6739	5.3931	3.0156	5.1579
MSE	0.0469	0.00018	8.5587	7.3794	16.4074	19.4322
RMSE	0.2166	0.0133	2.9255	2.7165	4.0506	4.4082
MAE	0.1783	0.0128	2.6570	2.1337	3.5316	4.0636
MAPE %	14.18	44.30	11.86	4.13	1.71	3.39

## Data Availability

The raw data supporting the conclusions of this article will be made available by the authors upon request. MATLAB code and ANN data-partition information will also be provided upon reasonable request.

## Abbreviations

The following abbreviations are used in this manuscript:

ANN	Artificial neural network
ASTM	American Society for testing and materials
ATR	Attenuated total reflectance
BR	Bayesian Regularization
FTIR	Fourier transform infrared spectroscopy
LM	Levenberg–Marquardt
MAE	Mean absolute error
MAPE	Mean absolute percentage error
MDI	Methylenediphenyl diisocyanate
MSE	Mean squared error
MSO	Modified soybean oil
RMSE	Root mean square error
SCG	Scaled conjugate gradient
PUB	Polyurethane-based biocomposite
TLS	Thermal conductivity measurement system
WAS	Waste artichoke stem

## References

[1] Maleki P., Akhavan-Safar A., Carbas R.J., Marques E.A., da Silva L.F. (2026). A comprehensive review of failures in bi-material interfaces: Mechanisms, prediction, and mitigation strategies. Proc. Inst. Mech. Eng. Part L J. Mater. Des. Appl..

[2] Hjiri M., Mustapha N., Benamara M. (2026). Advanced functional materials for photonic and extreme-environment applications—Structure–property–performance relationships. J. Mater. Sci..

[3] Issaka E., Melville L., Fazal A. (2026). Functional polymer composites for biomedical innovation: A review on classification, fabrication and future applications. Int. J. Polym. Sci..

[4] Shelly D., Singhal V., Jaidka S., Banea M.D., Lee S.Y., Park S.J. (2025). Mechanical performance of bio-based fiber reinforced polymer composites: A review. Polym. Compos..

[5] Kaufmann J., Temesgen A.G., Cebulla H. (2025). A comprehensive review on natural fiber reinforced hybrid composites processing techniques, material properties and emerging applications. Discov. Mater..

[6] Nautiyal S., Dimri S., Riyal I., Sharma H., Dwivedi C. (2025). Natural fibres and their composites: A review of chemical composition, properties, retting methods, and industrial applications. Cellulose.

[7] Nehhal S., Ali M.B., Abrouki Y., Ofqir K., Elkahoui Y., El Hajjaji S. (2025). Sustainable conversion of agricultural waste to activated biochar: Optimization and modelling of methylene blue adsorption. Ecol. Eng. Environ. Technol..

[8] Olcay H., Kocak E.D. (2022). The mechanical, thermal and sound absorption properties of flexible polyurethane foam composites reinforced with artichoke stem waste fibers. J. Ind. Text..

[9] Shibly M.A.H., Arman M.H., Sabur M.A., Bhuiyan M.A.H. (2024). Agro-waste from Gladiolus hybrida plants: Effects of alkaline processing on a new natural cellulosic fiber derived for polymer composites. ACS Sustain. Resour. Manag..

[10] Adam H.M., Bogale T.M., Paramasivam V. (2026). Natural fiber composites under dynamic loading: A comprehensive review of mechanical performance and structural integrity. Eng. Rep..

[11] Tanasă F., Zănoagă M., Teacă C.A., Nechifor M., Shahzad A. (2020). Modified hemp fibers intended for fiber-reinforced polymer composites used in structural applications—A review. I. Methods of modification. Polym. Compos..

[12] Shifa S.S., Zhang N. (2026). Progress and perspectives on plant-based natural fiber-reinforced polymer composites: Structure–property relationships and emerging design strategies. J. Polym. Res..

[13] Kaur R., Singh P., Tanwar S., Varshney G., Yadav S. (2022). Assessment of bio-based polyurethanes: Perspective on applications and bio-degradation. Macromol.

[14] Pfister D.P., Xia Y., Larock R.C. (2011). Recent advances in vegetable oil-based polyurethanes. ChemSusChem.

[15] Periyappillai G., Sathiyamurthy S., Saravanakumar S. (2024). Optimized machine learning prediction and RSM optimization of mechanical properties in boiled eggshell filler-added biocomposites. Fibers Polym..

[16] Aydoğmuş E. (2022). Biohybrid nanocomposite production and characterization by RSM investigation of thermal decomposition kinetics with ANN. Biomass Convers. Biorefin..

[17] Mulenga T.K., Rangappa S.M., Siengchin S. (2025). Natural fiber composites: A comprehensive review on machine learning methods. Arch. Comput. Methods Eng..

[18] Tanase M., Bolboaca R., Veres C. (2026). Machine learning in sustainable fiber-reinforced polymers: A bibliometric and critical assessment. Polym. Bull..

[19] Nasri K., Toubal L. (2024). Artificial neural network approach for assessing mechanical properties and impact performance of natural-fiber composites exposed to UV radiation. Polymers.

[20] Malashin I., Tynchenko V., Gantimurov A., Nelyub V., Borodulin A. (2024). A multi-objective optimization of neural networks for predicting the physical properties of textile polymer composite materials. Polymers.

[21] Ustaoğlu A., Menbari S., Gencel O., Aydoğmuş E., Sarı A., Yeşilata B., Ozbakkaloglu T., Uzun O. (2025). Development and characterization of coconut oil-based phase change material integrated flexible polyurethane biocomposites for thermal energy storage applications. Sol. Energy Mater. Sol. Cells.

[22] Bayram M., Aydoğmuş E., Gencel O., Ustaoğlu A., Sarı A., Hekimoğlu G., Memiş S., Yaprak H., Ozbakkaloglu T. (2026). Advancing energy savings and CO_2_ emission reductions in lightweight concrete with bio-based polyurethane phase change material for sustainable building applications. Cem. Concr. Compos..

[23] Aydoğmuş E., Yanen C., Kıstak C. (2025). Advancing sustainable materials: Synthesis and analysis of polyurethane biocomposites from hydrogenated safflower oil. Appl. Sci..

[24] Dağ M., Aydoğmuş E., Yalçın Z.G., Arslanoğlu H. (2023). Diatomite reinforced modified safflower oil-based epoxy biocomposite production: Optimization with RSM and assessment of outcomes by ANN. Mater. Today Commun..

[25] (2020). Standard Test Methods for Density and Specific Gravity (Relative Density) of Plastics by Displacement.

[26] Şahal H., Aydoğmuş E., Arslanoğlu H. (2025). Thermophysical properties and optimization of modified palm oil-amine reinforced biocomposites for lightweight and insulating applications. Mater. Sci. Eng. B.

[27] Dağ M., Aydoğmuş E., Yalçın Z.G., Arslanoğlu H. (2026). Valorization of industrial waste in polymer composites: Enhancing mechanical and thermal properties for insulation applications using machine learning analysis. Polym. Eng. Sci..

[28] (2021). Standard Test Method for Rubber Property—Durometer Hardness.

[29] (2022). Standard Test Method for Tensile Properties of Plastics.

[30] Buran A., Durğun M.E., Aydoğmuş E., Topdemir A. (2026). Optimization and characterization of epoxidized palm oil-based epoxy polymers: Structure–property relationships for sustainable material applications. J. Appl. Polym. Sci..

[31] Aydın M., Demirel M.H., Aydoğmuş E. (2026). Development and characterization of sustainable epoxy biocomposites reinforced with coconut shell powder and GNP. Polymers.

[32] (2022). Standard Test Methods for AC Loss Characteristics and Permittivity (Dielectric Constant) of Solid Electrical Insulation.

[33] Demirel M.H., Aydoğmuş E. (2022). Waste polyurethane reinforced polyester composite, production, and characterization. J. Turk. Chem. Soc. Sect. A Chem..

[34] Orhan R., Aydoğmuş E. (2022). Investigation of some thermophysical properties of *Asphodelus aestivus* reinforced polyester composite. Fırat Univ. J. Exp. Comput. Eng..

[35] Aydın M., Aydoğmuş E., Arslanoğlu H. (2025). Comparative analysis of artificial neural networks and adaptive neuro-fuzzy inference system for biocomposite material synthesis and property prediction. Mater. Chem. Phys..

[36] Sambath Y., Natarajan R., Babu P.K., Raju K.R., Alahmadi A.A., Alwetaishi M., Khan S.A. (2025). Comparative analysis of predictive modeling techniques for mechanical properties in dissimilar friction stir welding of AA6061 and AZ31B. J. Mater. Eng. Perform..

[37] Huang Y., Wu Q., Gong T., Yang J., Luo Q., Liu M. (2025). Bayesian neural network with unified entropy source and synapse weights using 3D 16-layer Fe-diode array. Nat. Commun..

[38] Ogunsola N.O., Shin C., Lawal A.I., Kim Y.K., Cho S. (2025). Blasting-induced ground vibration modeling in tunnel excavation: A comparative study of ANN, hybrid ANNs, and empirical models. Rock Mech. Rock Eng..

[39] Jafari A., Mollaali M., Ma L., Shahmansouri A.A., Zhou Y., Dugnani R. (2025). Brittle fracture strength prediction via XML with reliability considerations. Eng. Fract. Mech..

[40] Jafari A., Ma L., Shahmansouri A.A., Dugnani R. (2023). Quantitative fractography for brittle fracture via multilayer perceptron neural network. Eng. Fract. Mech..

[41] Yu H., Wilamowski B.M. (2018). Levenberg–Marquardt training. Intelligent Systems.

[42] Sariev E., Germano G. (2020). Bayesian regularized artificial neural networks for the estimation of the probability of default. Quant. Financ..

[43] Solairaju J.A., Rathinasamy S., Thanikodi S., Tarawneh B., Gurumoorthy V., Antony J.S., Arul Gnana Dhas A. (2025). Development and validation of ANN models for water absorption in sawdust and kenaf fiber-reinforced polystyrene composites. Eng. Rep..

[44] Shettar M., Bhat A., Katagi N.N., Gowrishankar M.C., Somdee P. (2026). Sustainable optimization of mechanical properties in fiber-reinforced polymer composites using RSM and ANN: A systematic review. J. Mater. Res. Technol..

[45] Laabid Z., Lakhdar A., Mansouri K., Siadat A. (2024). Implementation of artificial intelligence in the prediction of the elastic characteristics of bio-loaded polypropylene with bamboo fibers. Int. J. Electr. Comput. Eng..

[46] Long T., Pang Q., Deng Y., Pang X., Zhang Y., Yang R., Zhou C. (2025). Recent progress of artificial intelligence application in polymer materials. Polymers.

[47] Dagdag O., El Gana L., Haldhar R., Berisha A., Kim S.-C., Berdimurodov E., Hamed O., Jodeh S., Akpan E.D., Ebenso E.E. (2023). Study on thermal conductivity and mechanical properties of cyclotriphosphazene resin-forced epoxy resin composites. Crystals.

[48] Wang B., Zhao F., Xu K., Wen T., Jiang L. (2023). An efficient multi-parameter synchronous identification method for fiber-reinforced laminated structure based on improved Levenberg–Marquardt algorithm and modal data. J. Vib. Eng. Technol..

[49] Carlone P., Aleksendrić D., Ćirović V., Palazzo G.S. (2014). Meta-modeling of the curing process of thermoset matrix composites by means of a FEM–ANN approach. Compos. Part B Eng..

[50] Rahman S.K., Al-Ameri R. (2023). Structural assessment of basalt FRP reinforced self-compacting geopolymer concrete using artificial neural network (ANN) modelling. Constr. Build. Mater..

[51] Çolak A.B. (2021). An experimental study on the comparative analysis of the effect of the number of data on the error rates of artificial neural networks. Int. J. Energy Res..

[52] Bafitlhile T.M., Li Z., Li Q. (2018). Comparison of Levenberg–Marquardt and conjugate gradient descent optimization methods for simulation of streamflow using artificial neural network. Adv. Ecol. Environ. Res..

[53] Zhang Z., Friedrich K. (2003). Artificial neural networks applied to polymer composites: A review. Compos. Sci. Technol..

[54] Hasan M.M., Rahman M.M., Abu Bakar S., Kabir M.N., Ramasamy D., Saifullah Sadi A.H.M. (2025). Performance evaluation of various training functions using ANN to predict the thermal conductivity of EG/water-based GNP/CNC hybrid nanofluid for heat transfer application. J. Therm. Anal. Calorim..

[55] Ahmad N., Nath V. (2025). A systematic review of machine learning-based small-signal modeling approaches for gallium nitride high electron mobility transistors: Performance analysis and algorithmic insights. Indian J. Pure Appl. Phys..

[56] Mairpady A., Mourad A.H.I., Mozumder M.S. (2021). Statistical and machine learning-driven optimization of mechanical properties in designing durable HDPE nanobiocomposites. Polymers.

[57] Sienkiewicz N., Dominic M., Parameswaranpillai J. (2022). Natural fillers as potential modifying agents for epoxy composition: A review. Polymers.

[58] Capretti M., Giammaria V., Santulli C., Boria S., Del Bianco G. (2023). Use of bio-epoxies and their effect on the performance of polymer composites: A critical review. Polymers.

[59] Monticeli F.M., Neves R.M., Ornaghi H.L., Almeida J.H.S. (2022). Prediction of bending properties for 3D-printed carbon fibre/epoxy composites with several processing parameters using ANN and statistical methods. Polymers.

